# G-quadruplex-binding ligand RHPS4 reduces *Plasmodium falciparum* mitochondrial DNA level providing a novel antimalarial strategy

**DOI:** 10.1128/aac.00144-26

**Published:** 2026-10-01

**Authors:** Mariam Salim, Lucie Paloque, Flore Nardella, Thibaud Reyser, Michel Nguyen, Jean-Michel Augereau, Yu Luo, Sébastien Britton, Jean-Louis Mergny, Virginie Gervais, Françoise Benoit-Vical, Dennis Gomez

**Affiliations:** 1 IPBS, CNRS, Université de Toulouse, Toulouse, France; 2 Université de Toulouse, CNRS, Inserm, LCC, ERL 1289 MAAP, Toulouse, France; 3 Laboratoire d’Optique et Biosciences, Ecole Polytechnique, CNRS, Inserm, Institut Polytechnique de Paris 600094, Palaiseau, France; 4 CNRS UMR9187, INSERM U1196, Institut Curie, PSL Research University, Orsay, France; 5 Université Paris-Saclay, CEA, CNRS - Institute for Integrative Biology of the Cell (I2BC) 27048, Gif-sur-Yvette, France; The Children's Hospital of Philadelphia, Philadelphia, Pennsylvania, USA

**Keywords:** G-quadruplex, G4 ligands, new antiplasmodial drug, *Plasmodium*, malaria

## Abstract

Malaria caused by *Plasmodium falciparum* remains a major health threat, killing over 600,000 people annually. The spread of resistance to all major antimalarials, including artemisinins, highlights the urgent need for new drugs with distinct mechanisms of action. Here, we show that the G-quadruplex ligand RHPS4, an acridine derivative, displays strong antiplasmodial activity against both drug-sensitive and drug-resistant *P. falciparum* strains and clinical isolates. RHPS4 primarily targets the trophozoite stage and induces major mitochondrial alterations, including reduction of mitochondrial DNA (mtDNA) and transcriptional dysfunctions. Bioinformatic analyses identified at least eight putative G4-forming sequences within the parasite’s mtDNA. Biophysical studies confirmed G4 folding of at least one sequence and its interaction with RHPS4. These findings indicate that RHPS4 impacts *P. falciparum* mitochondrial DNA replication and transcription, leading to parasite death, and establish G4 ligands as novel antimalarial agents.

## INTRODUCTION

*Plasmodium falciparum* malaria still kills yearly more than 600,000 people, the vast majority of whom are children under the age of 5 years, especially in sub-Saharan Africa (1). In recent decades, malaria treatment has relied on fewer than a dozen antiplasmodial drugs, which mainly target heme polymerization (e.g., chloroquine), pyrimidine biosynthesis (e.g., sulfadoxine-pyrimethamine), cytochrome b activity (e.g., atovaquone), or lead to heme and protein alkylation (e.g., artemisinin derivatives) (2). Unfortunately, *P. falciparum* has always been able to develop resistance to these drugs, including to the current major antimalarial drug, artemisinin (ART), and its derivatives (3, 4). ART resistance is emerging and spreading in Africa (5), where 90% of malaria cases occur, raising fears of a therapeutic dead end in the medium term. Now, the challenge is to develop drugs with novel modes of action to limit the risk of cross-resistance with existing drugs. Several new targets are under investigation, including ATPase 4, phosphatidylinositol-4-kinase, eukaryotic elongation factor 2, histone deacetylase 1, acetyl-CoA synthetase, and the proteasome (6). Guanine quadruplexes (G4, four-stranded non-canonical secondary structures of guanine-rich DNA or RNA sequences) (7, 8) have been considered as potential targets to counteract parasite proliferation, and their selective stabilization by small chemical compounds, known as G4 ligands (9), has been shown to exert effects that, although modest, remain of significant interest (10–15). Over the past years, numerous studies have demonstrated the essential role of G4 in cell survival and cell metabolism regulation in different models (16, 17). In cells, the stabilization of DNA-G4 structures by G4 ligands drives genomic instability, which is characterized by DNA breaks, DNA deletions, and chromosomal translocations (18–24). The formation of DNA-G4 structures is not restricted to the nuclear compartment. In human cells, G4 structures have been associated with mitochondrial DNA deletion breakpoints and with the stalling of the replication fork during mitochondrial DNA replication (25, 26), contributing to the regulation of mitochondrial gene expression and to perturbations of the mitochondrial genome (27–29). The presence of G4 structures in *Plasmodium* has been confirmed with specific G4-detecting antibodies (13, 15). Given that *P. falciparum* genome is characterized by its extremely AT-rich genome (>80%) (30), only a few regions of the parasite genome have the potential to form G4 structures (10, 13, 14, 31–33). Interestingly, a genome-wide analysis of the nuclear and mitochondrial DNA of *Plasmodium* shows a higher density of G4 motifs in the mitochondrial DNA compared with the nuclear DNA (31).

To our knowledge, the impact of G4 ligands on *P. falciparum* mitochondrial genome has not yet been evaluated. Among several compounds already described as G4 ligands in mammalian cells, the acridine derivative RHPS4 was identified as a selective telomerase inhibitor through its capacity to bind and fold telomeric sequences into G4 (34). RHPS4 provokes telomeric dysfunctions and replication stress, induced by its capacity to impair fork progression in G4-containing regions in human transformed fibroblasts and melanoma cells (35–37). While most cellular studies performed with RHPS4 have focused on its telomeric effects, Falabella and co-workers reported that RHPS4 preferentially localizes into mitochondria where it abolishes mitochondrial functions and alters both mitochondrial replicative and transcriptional mechanisms (28).

In this report, we show that RHPS4 exhibits a substantial antiplasmodial activity against *P. falciparum* parasites, including on various drug-resistant laboratory strains and clinical isolates. We show that RHPS4 targets the trophozoite stage and induces important mitochondrial changes in *Plasmodium,* such as a strong reduction in mtDNA content coupled to transcriptional dysfunctions. Using bioinformatics analyses, we identified eight putative G4-forming sequences in the mitochondrial genome of the *P. falciparum* parasite, and G4 folding of one sequence was experimentally confirmed. Furthermore, we demonstrated G4 interaction with RHPS4. Our study highlights RHPS4 as a promising antiplasmodial compound with a novel mechanism of action. These results open new avenues for antimalarial drug discovery targeting mitochondrial nucleic acid structures and underscore the potential of G4 ligands in combating drug-resistant malaria.

## RESULTS

### RHPS4 has a potent and selective antiplasmodial activity

**Fig 1:**
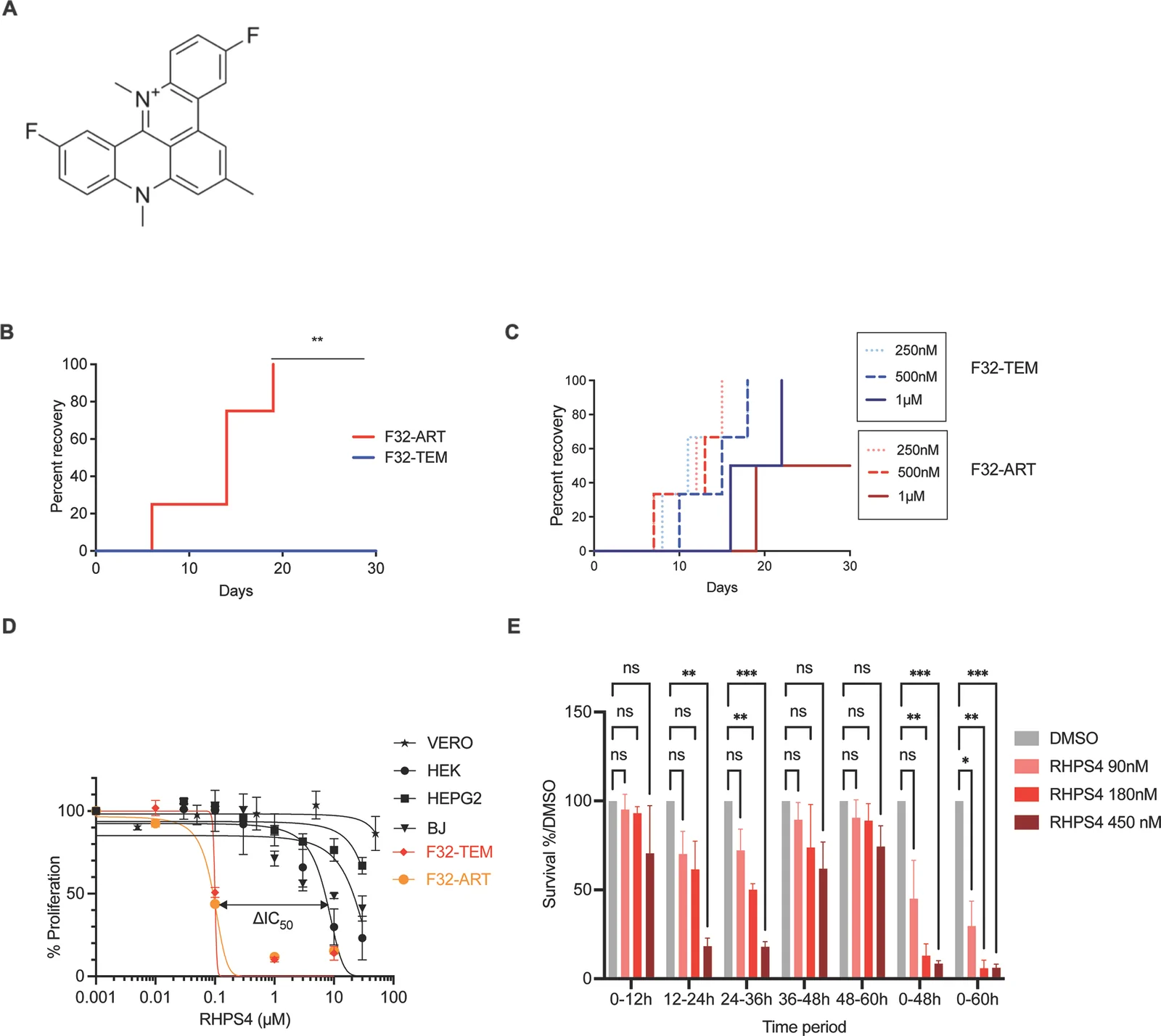
Selective antiplasmodial activity of the G-quadruplex ligand RHPS4. (**A**) Chemical structure of RHPS4. (**B and C**) Kaplan-Meier analyses of *P. falciparum in vitro* recrudescence tests comparing the ability of F32-ART (red color gradients) and F32-TEM (blue color gradients) strains to proliferate after treatments: (**B**) ART 18 µM as control, (**C**) RHPS4 250, 500, and 1,000 nM. The Mantel-Cox test was used for statistical analysis of the recrudescence data. (**p < 0.01). (**D**) Selectivity of RHPS4 toward parasites *versus* human cells. HEK, HepG2, and BJ-hTERT cell lines were treated for 72 h with a concentration range of RHPS4, and viability was measured by the sulforhodamine B assay while Vero cells were treated for 48 h and viability was measured by MTT to quantify cell metabolic activity. The selectivity index (SI) denotes the ratio between the CC₅₀ of the most sensitive human line (HEK) and the IC₅₀ of each *P. falciparum* strain. (**E**) Stage-specific activity of RHPS4. The F32-TEM strain was treated with RHPS4 using the IC₅₀ concentration, 2  ×  IC₅₀, or 5  ×  IC₅₀, initiated at successive 12-h intervals during five successive 12-h periods from a highly synchronized ring stage population. Following each pulse, parasites were washed and cultured drug-free until 60 h. The % of survival was then determined using stained blood smear and normalized to DMSO treatment. Error bars indicate mean  ±  SD (*n*  =  3 independent experiments).*p < 0.05; **p < 0.01; ***p < 0.001; ns denotes a non-significant difference.

The antiplasmodial activity of five chemically diverse G4 ligands (34, 38–41) was assessed *in vitro* on different *P. falciparum* strains. Strikingly, only RHPS4 (Fig. 1A), with an IC_50_ value of 90 ± 10 nM, showed a strong antiplasmodial activity against the drug-sensitive strain F32-TEM (Table 1), ranging in the same order of magnitude as the three major antimalarial drugs ART, chloroquine (CQ), and atovaquone (ATQ).

**TABLE 1:** IC₅₀ values of G-quadruplex ligands and reference antiplasmodial drugs against *Plasmodium falciparum* strains^*a*^^,^ ^*d*^

Compounds	IC_50_ ± SD (nM)						
	F32-TEM	F32-ART	IPC 8262	Q120	3D7^*c*^	RF12^*c*^	Dd2^*c*^
RHPS4	90 ± 10	80 ± 20	42 ± 2	48 ± 6	26	36 ± 11	42 ± 1
360A	730 ± 150	770 ± 200	n.d	n.d	n.d	n.d	n.d
PhenDC3	1090 ± 420	1250 ± 470	n.d	n.d	n.d	n.d	n.d
Pyridostatin	3770 ± 1000	2700 ± 250	n.d	n.d	n.d	n.d	n.d
CX-5461	2270 ± 460	1930 ± 60	n.d	n.d	n.d	n.d	n.d
Artemisinin	26 ± 5	30 ± 10	18 ± 10	1.9 ± 1.0	65	0.8	0.6
Atovaquone	1.3 ± 0.1	0.8 ± 0.5	1.0 ± 0.1	4870 ± 375	0.4	n.d	n.d
Chloroquine^*b*^	35 ± 1	35 ± 4	111 ± 28	450	n.d	n.d	n.d

^*a*^ IC₅₀ values of various G4 ligands, and reference antiplasmodial drugs (ART, artemisinin; ATQ, atovaquone; CQ, chloroquine) on several *Plasmodium falciparum* strains: F32-TEM (drug-sensitive), F32-ART (ART-resistant), IPC8262 (resistant to both ART and CQ), and Q120 (resistant to both ATQ and CQ). IC₅₀ values were determined based on DNA quantification after 48 h of treatment with a concentration range for each compound. Data represent the mean of five independent experiments (*n* = 5), except for 3D7 (drug sensitive, *n* = 1), RF12 (CQ-resistant and ART-resistant, *n* = 2), and Dd2 (CQ-resistant, *n* = 2).

^*b*^ These values have been previously published in reference 42.

^*c*^ Data from Medicine for Malaria Venture using Pf LDH assay and 72-h incubation.

^*d*^ n.d., not determined.

We next evaluated all compounds against the ART-resistant strain F32-ART (43). As previously observed, most of the G4 ligands remained weakly active, while RHPS4 maintained a strong antiproliferative effect against the F32-ART strain, with an IC_50_ value of 80 ± 20 nM. This activity was also similar in the multidrug-resistant clinical field isolates IPC8262 (resistant to ART and CQ), and Q120 (resistant to ATQ and CQ) with IC_50_ values of 42 ± 2 nM and 48 ± 6 nM, respectively, showing no cross-resistance between RHPS4, CQ, and ATQ (Table 1). These results were confirmed in another laboratory with a different assay against three other strains (3D7, RF12, and Dd2), as part of a collaboration with Medicines for Malaria Venture (MMV) (Table 1). Due to the quiescence-based mechanism of ART resistance (43), IC_50_ determination cannot provide evidence of cross-resistance with ART, which is assessed through a recrudescence assay based on the difference between ART-resistant and ART-sensitive parasites in their ability to proliferate after treatment (44). As expected, when treated with ART 18 μM for 48 h, the ART-resistant F32-ART strain was able to resume its growth after drug washout (median recovery time of 14 days), while no ART-sensitive parasites survived (no recrudescence of F32-TEM at day 30) (Fig. 1B). Using the same assay, the recrudescence capacities of the two strains F32-TEM and F32-ART were assessed after treatment with 250 nM, 500 nM, and 1 µM of RHPS4 (corresponding to 3, 6, and 12 times the IC_50_ value of RHPS4). While a short delay in recrudescence was observed with 1 µM of RHPS4, the recrudescence capacities of the two strains F32-TEM and F32-ART were not statistically different at all concentrations (*P*-value = 0.69, 0.78, and 0.43 for 250 nM, 500 nM, and 1 µM, respectively), demonstrating the absence of cross-resistance between ART and RHPS4 (Fig. 1C). An important point for antimalarial drugs profile is their selective activity against parasites *versus* the host cells. This was evaluated using cytotoxicity assays on four different mammalian cell lines: HepG2 (derived from hepatocarcinoma), HEK (derived from embryonic kidney), BJ-hTERT cells (immortalized non-transformed human fibroblast), and Vero cells (isolated from monkey kidney epithelial cells). CC_50_ values of RHPS4 towards human and monkey cells were close to or higher than 10 µM (Fig. 1D), indicating a highly selective action of RHPS4 against *P. falciparum* parasites compared with mammalian cells with a selectivity index (SI) > 100.

### RHPS4 is active during the trophozoite stage

During its 48-h erythrocytic cycle, *P. falciparum* matures and multiplies through different stages: ring, trophozoite, and schizont. This ultimately leads to the release of new parasites into the bloodstream, initiating a new cycle. To evaluate the stage-specificity of RHPS4 action, we determined its activity at different concentrations during five successive 12-h periods from a highly synchronized ring-stage population. As a control, we treated the parasites for 48 and 60 h. Consistent with our IC_50_ results, a 50% inhibitory effect was observed after 48 h of treatment with 90 nM of RHPS4 (IC_50_ value). The parasite survival was affected by increasing both the concentration and the incubation time. The impact of RHPS4 on *P. falciparum* proliferation is markedly stage-dependent. Indeed, a minor impact on parasitemia was noted for 12-h treatments initiated at 0, 36, and 48 h, even at the highest RHPS4 concentration. In contrast, a pronounced effect on survival was observed when the 12-h treatment was initiated at 12 or 24 h, with a concentration-dependent effect of RHPS4 (Fig. 1E).

Overall, our results indicate that RHPS4 acts mainly during the 12–36-h period of the parasite's asexual blood cycle, which coincides with the trophozoite stage.

### RHPS4 is slow acting against *P. falciparum* parasites

**Fig 2:**
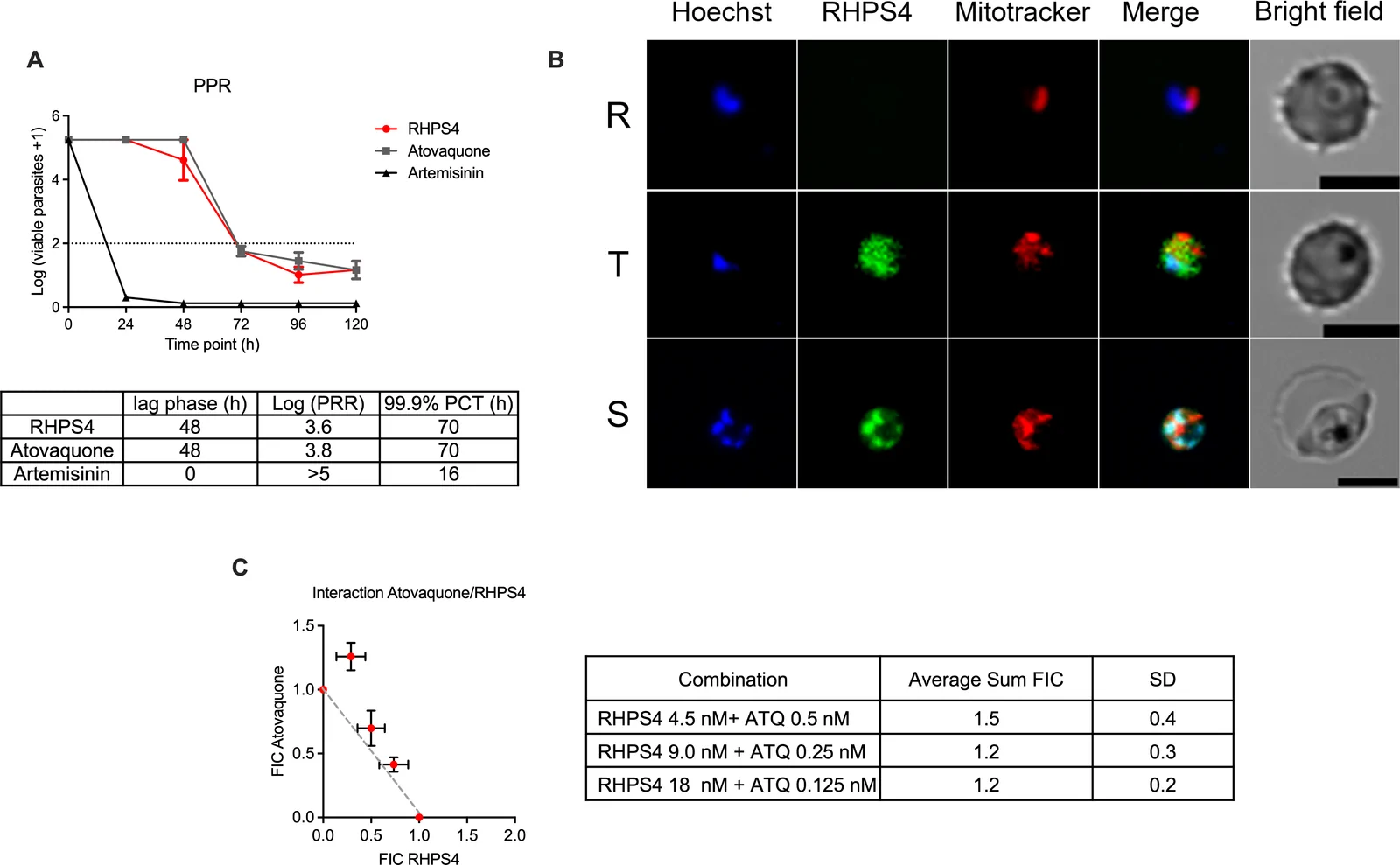
Mitochondrial effects, and drug interaction profile of RHPS4 in *Plasmodium falciparum*. (**A**) Determination of the parasite reduction ratio (PRR): *in vitro* lag phase, parasite reduction ratio, and 99.9% parasite clearance times (PCT) in response to RHPS4, atovaquone, and artemisinin exposures at a concentration corresponding to 10× IC_50_. Lag phase: time needed to achieve the maximum rate of killing. Log (PRR): log10 of parasite reduction ratio over one cycle (48h) when maximum speed of action is achieved. 99.9% PCT: time required to reduce 99.9% of the initial parasite population. (**B**) Confocal microscopy images of parasites treated for 30 min with the nuclei acid strain Hoechst 33342 (blue), 450 nM RHPS4 (green), and 100 nM Mitotracker Deep Red FM (red) to label mitochondria at the stage of R = ring, T = trophozoite, S = schizont. Scale bar corresponds to 5 µm. More images are available in Fig. S1. (**C**) Isobologram and sum of fractional IC_50_ (FIC) of the interaction between RHPS4 and ATQ on *P. falciparum* F32-TEM strain. Error bars represent the standard deviation of four independent experiments. In the case of an additive combination of the two molecules, the isobologram lies on a straight line. In the case of a synergistic combination, the isobologram lies below the line, while in the case of antagonistic combinations, it lies above it. The FIC is the ratio of the IC_50_ of the combination to the IC_50_ of the molecule alone. More precisely, ΣFICs <1 denote synergism, ΣFICs ≥1 and <2 denote additive interaction, ΣFICs ≥2 denote antagonism, and ΣFICs ≥4 denote marked antagonism (45).

The speed of action of a compound determines the exposure time required to achieve antiplasmodial activity. We determined the parasite reduction ratio (PRR) of *P. falciparum* F32-TEM strain in response to treatment with 10 times the IC_50_ of RHPS4 for up to 120 h, using ATQ and ART as controls. RHPS4 showed a slow-acting parasite reduction profile similar to ATQ with a time to achieve the maximum speed of action (lag phase) of 48 h, a parasite reduction ratio of 3.6 log over 48 h [Log(PRR)], and a 99.9% parasite clearance time (PCT) of 70 h (Fig. 2A). On the contrary, the fast-acting compound ART presented a 99.9% PCT value of 16 h with no lag phase and a Log(PRR) value greater than 5 (extrapolated from the first 24 h of treatment) (Fig. 2A). These data were confirmed by monitoring the parasite morphology throughout the treatment period (Fig. S1A). In the control condition (DMSO), reinvasion was observed every 48 h (full cycle), as evidenced by the rings observed corresponding to the beginning of a second cycle. In contrast, after 72 h of continuous exposure to RHPS4 or ATQ, intraerythrocytic development was blocked at the trophozoite stage. These microscopic observations indicate that RHPS4 and ATQ achieve antiplasmodial activity at the trophozoite stage and confirm the PRR data with a lag phase of 48 h. The rapid onset of action of ART is also confirmed by the fact that, after 24 h of continuous exposure to artemisinin, parasites are dead with only pyknotic parasites visible in the smear.

These results show that RHPS4 is a slow-acting compound, similarly to ATQ, a well-known inhibitor of *P. falciparum* mitochondrial metabolism targeting cytochrome b (46). This contrasts with the fast-acting ART compound, which acts *via* an iron-dependent mechanism leading to ROS production.

### RHPS4 impacts *P. falciparum* mitochondrial metabolism

Initially described as a selective telomerase inhibitor and shown to alter telomeric functions, RHPS4 has also been reported to impair mitochondrial metabolism in mammalian cells (28). Mitochondrial dysfunctions induced by RHPS4 appear at low concentrations (nanomolar range), with no impact on nuclear DNA, indicating a preferential accumulation in the mitochondrial organelle (28). Based on these observations, we investigated the subcellular localization of RHPS4 in *Plasmodium falciparum,* in the different asexual blood stages, using Hoechst 33342 for DNA staining, MitoTracker Deep Red FM for mitochondrial staining, and RHPS4 at 450 nM. This concentration was chosen as the fluorescence signal of RHPS4 is not detectable at a concentration of 90 nM (corresponding to the IC_50_ in *P. falciparum*), even after 48 h of treatment. At higher concentration, we observed accumulation of RHPS4 within the parasite, with a stage-dependent localization pattern characterized by the absence of detectable fluorescence in ring-stage parasites, a pan-parasite distribution in trophozoites, and a mainly nuclear localization in schizonts (Fig. 2B; Fig. S1B). Notably, RHPS4 parasite localization correlates with its moment of action (Fig. 1E) since no activity was observed in rings, low activity at the schizont stage, and peak activity at the trophozoite stage when fluorescent signal of RHPS4 is observed in the whole parasite.

These findings are consistent with previous observations by Heald et al., who reported that, at high concentrations, RHPS4 localizes to the cytoplasm, nuclear membrane, and nucleoli of MCF-7 cells (47).

### Additive effect of RHPS4 with atovaquone

As RHPS4 seems to affect mitochondria, we studied its interaction with ATQ. As a control, we tested the combination ATQ + proguanil as this synergistic combination is used in therapy. As expected, we obtained a synergistic effect with sums of fractional IC_50_ (FIC) values below 1, consistent with a previous study (Fig. S1C). By contrast, the combination of ATQ with RHPS4 shows an additive effect, as the sums of the fractional IC_50_ values are close to 1 (Fig. 2C).

### RHPS4 alters nucleic acid transactions in *Plasmodium* mitochondria

**Fig 3:**
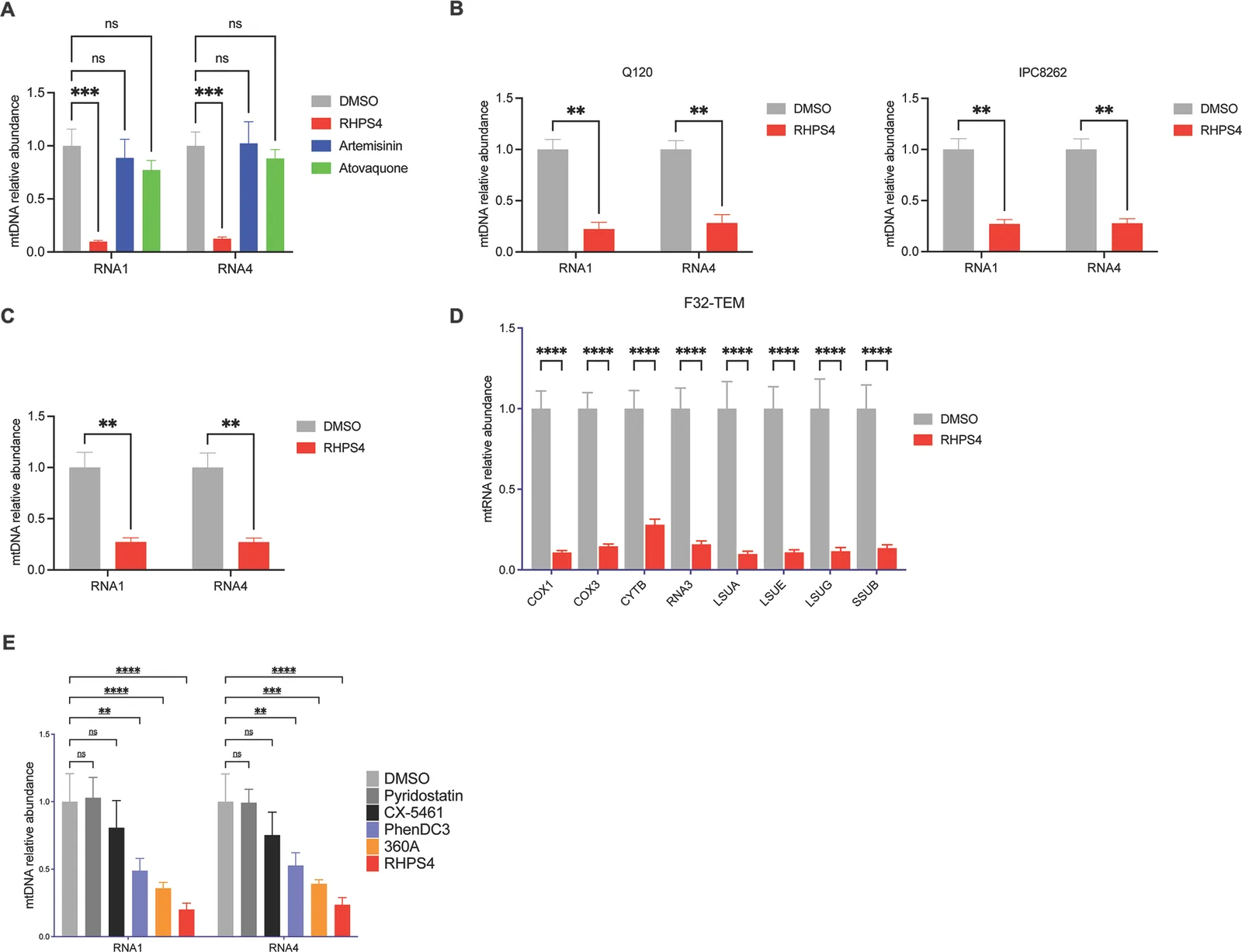
RHPS4 alters mitochondrial DNA abundance in *Plasmodium falciparum* and clinical isolates. (**A–C**) Relative abundance of different mtDNA sequences at the trophozoite stage, measured by qPCR after a 48-h exposure to RHPS4, artemisinin, and atovaquone at their respective IC₅₀ values (90, 20, and 5 nM, respectively) using the F32-TEM strain (**A**), Q120, and IPC8262 (**B**). For both clinical isolates, which present a slightly slower replicative cycle than laboratory strains, data were obtained after a 72-h treatment. Bars show mean ± SD from *n* = 3 independent experiments for F32-TEM and from *n* = 2 independent experiments for both clinical isolates. (**C**) Relative abundance of mtDNA sequences was measured by qPCR following a 24-h exposure to 180 nM RHPS4 in highly synchronized F32-TEM parasites (12–16 h post-invasion). Bars represent mean ± SD from three independent experiments (*n* = 3). (**D**) Relative abundance of transcripts (mRNA) encoded by mtDNA measured by RT-qPCR following a 24-h exposure to 180 nM RHPS4 in highly synchronized F32-TEM parasites (12–16 h post-invasion). Bars represent mean ± SD from three independent experiments (*n* = 3). (**E**) Relative abundance of mtDNA sequences measured by qPCR after 48 h of exposure to different G4 ligands at their respective IC₅₀ concentrations. Bars show mean  ±  SD from *n*  =  3 independent experiments. For all experiments, mtDNA abundance was normalized to the nuclear gene *PF3D7_1371000*, encoding 18S ribosomal RNA. For RNA quantification, the nuclear transcript *PF3D7_1121300*, encoding the TKL2 protein, was used as the normalization control. **p < 0.01; ***p < 0.001; ****p < 0.0001. ns denotes a non-significant difference.

Because mitochondrial dysfunctions induced by RHPS4 in mammalian cells are associated with alterations in nucleic acid transactions, qPCR assays were performed to evaluate RHPS4 impact on F32-TEM mtDNA. The 6 kb *P. falciparum* mitochondrial genome encodes for many small ribosomal RNA subunits and for three essential components of the mitochondrial electron transport chain: cytochrome c oxidase subunit 1 (COX1), cytochrome c oxidase subunit III (COX3), and cytochrome b (CYTB). We extracted the total parasite genomic DNA after 48 h of exposure to 90 nM RHPS4 (corresponding to the IC_50_ value), starting with trophozoite parasites, and quantified the relative abundance of mtDNA (normalized to nuclear DNA) by qPCR with primers targeting different mtDNA regions (coding for ribosomal RNA fragments RNA1 and RNA4 and COX1, COX3, and CYTB coding regions, Table S1). Treatment with RHPS4 resulted in 5- to 10-fold reduction in mtDNA relative abundance compared with the DMSO control for all DNA regions tested (Fig. 3A; Fig. S2A). As controls, mtDNA abundance was evaluated on parasites treated with the two antimalarial drugs ART and ATQ. After 48 h treatment at their respective IC_50_ concentrations, no significant reduction was observed in mtDNA relative abundance compared with the DMSO condition (Fig. 3A). Importantly, RHPS4 did not interfere with the PCR assay itself, since the addition of up to 10 µM RHPS4 in the PCR mix did not impact, *in vitro,* mtDNA amplification (Fig. S2B). These results strongly suggest that RHPS4 operates through an original mechanism directly related to mtDNA, as compared with antimalarial drugs. A similar result, with a fivefold reduction in mtDNA abundance after RHPS4 treatment, was obtained with the field isolates Q120 and IPC8262 (Fig. 3B), indicating a shared mechanism of action of RHPS4 independent of the *P. falciparum* strain. Finally, we observed the same significant effect on mtDNA abundance when highly synchronized parasites (12–16 h post-invasion) were treated for 24 h with 180 nM of RHPS4, to cover the trophozoite stage, when RHPS4 is the most active (Fig. 1E), showing that RHPS4-induced mtDNA depletion correlated with its stage-specific activity (Fig. 3C). Based on these results, we hypothesized that the production of mitochondrial mRNAs and rRNAs should be equally affected by the RHPS4 treatment. RT-qPCR analyses were performed on the total RNA extracted from highly synchronized trophozoites treated with 180 nM RHPS4 for 24 h. RT-qPCR analyses showed a 5- to 10-fold reduction of all tested transcript levels in treated parasites relative to control, which is consistent with the depletion of mtDNA induced by RHPS4 (Fig. 3D), indicating a global impact of RHPS4 on *P. falciparum* mtDNA transcription. Considering both the essential role of the subcellular organelle apicoplast in parasite survival and the potential for its own genome to form G-quadruplex (G4) structures, we conducted qPCR analyses to assess the effect of RHPS4 on apicoplast DNA. qPCR experiments targeting two distinct regions of the apicoplast genome (tRNA Methionine: AP-1 and Chaperone protein CIpM: AP-2, Table S1) showed no significant changes in apicoplast DNA levels. Importantly, these analyses were performed on the same DNA samples that exhibited marked alterations in mitochondrial DNA (mtDNA) abundance, highlighting a differential effect of RHPS4 on the two organelles (Fig. S2C).

As shown in Table 1, RHPS4 exhibits a marked antiproliferative effect compared with other G-quadruplex ligands. To determine whether there is a correlation between the antiproliferative effects of these compounds and their ability to affect the mitochondrial genome of the parasite, comparative qPCR analyses were performed. As shown in Fig. 3E, pyridostatin and CX-5461 had no significant effect on mtDNA abundance following treatment at concentrations corresponding to their respective IC_50_ values. In contrast, under the same treatment conditions, PhenDC3 and 360A—both charged compounds—induced significant reductions in mtDNA content, by approximately 50% and 65% respectively, relative to the DMSO control. However, the reduction of mtDNA by these two compounds was significantly lower than the massive reduction (80%) achieved by RHPS4 (Fig. S2D). Altogether, these data suggest a positive correlation between the ability of different ligands to perturb the mitochondrial genome of the parasite and their antiproliferative activity.

### RHPS4 interacts with a G-quadruplex forming sequence from *P. falciparum* mtDNA

*In silico* analysis of *P. falciparum* mtDNA indicated the presence of at least eight sequences having the potential to form G-tetrad G4 structures (Table S2) (in order not to miss any G4 candidate, relatively permissive parameters were chosen to identify these motifs). Two of them, Pf_mtDNA_G4_1 and Pf_mtDNA_G4_4, were predicted to fold into a G4 by three different algorithms (Table 2).

**TABLE 2:** mtDNA sequence choice and summary of biophysical results^*a*^^,^^*c*^

QPS mtDNA		G4RNA screener v0.2					Biophysical results	
Sequence name	Sequence (5′ => 3′)	cGcC	G4H	G4NN	CD	TDS	IDS	Iso-FRET
Pf_mtDNA_G4_1	TGGATGGTGTTGGCTGGGC	11.50^*b*^	1.05^*b*^	0.93^*b*^	ap	Y	Y	Y
Pf_mtDNA_G4_2	CGGTAAGAAGGTTCGCCGGGGATAACAGGT	4.86^*b*^	0.77	0.79^*b*^	–	N	N	?
Pf_mtDNA_G4_3	AGGATTATTCGGAGGATTTGGT	16.00^*b*^	0.68	0.07	–	N	N	N
Pf_mtDNA_G4_4	TGGTGGTGGAACTGGATGGA	20.00^*b*^	0.95^*b*^	0.87^*b*^	–	N	N	?
Pf_mtDNA_G4_5	TGGAGGTACTACTGGAGTTATATTAGGTB	8.50^*b*^	0.54	0.03	–	N	N	N
Pf_mtDNA_G4_6	AGGGAAGTTAAGGTGCTCAGGGTCTTACCGTCGGGC	4.11	0.70	0.89^*b*^	–	N	N	?
Pf_mtDNA_G4_7	CGGCGGCTGTATTTTAAACGGTCCTAAGGT	2.13	0.30	0.03	–	N	N	N
Pf_mtDNA_G4_8	TGGAGCACTGGATTGGATACCCGGGA	1.92	0.42	0.42	–	N	N	N

^*a*^ Eight candidate DNA sequences were selected based on scores according to three different algorithms (cGcC, G4H for G4Hunter, and G4NN).

^*b*^ Scores above arbitrary thresholds are considered to be potential candidates and are indicated with a.

^*c*^ The biophysical results are summarized in the last four columns. For the CD spectra, “ap” stands for a antiparallel G4 structure; “–”: inconclusive. For TDS, IDS and iso-FRET, “Y,” “N,” and “?” stand for “Yes,” “No,” and “unconclusive,” respectively (whether the results are similar to what is observed for known G4-forming sequences).

**Fig 4:**
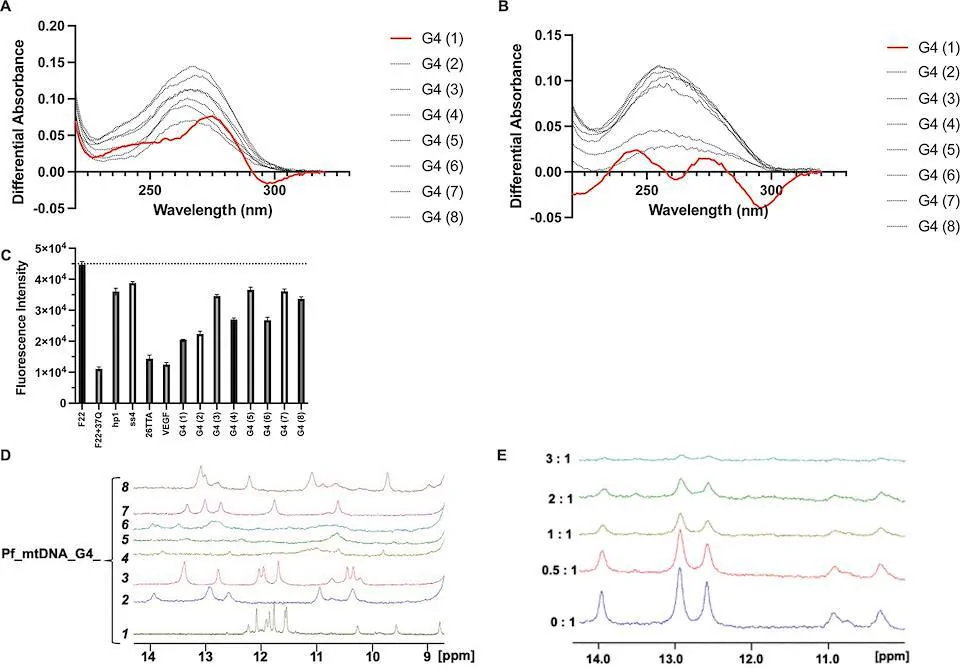
Biophysical characterization of G-quadruplex formation and RHPS4 binding to a mitochondrial DNA sequence of *Plasmodium falciparum*. (**A**) CD spectra result for the eight sequences listed in Table 1 (in the same order). The only sequence for which CD results are clearly indicative of G4 formation is shown in red (Pf_mtDNA_G4_1). (**B**) Evaluation of G4 formation by NMR. A first spectrum was recorded at 278K for Pf_mtDNA_G4_1 (300 mM concentration) dissolved in a cacodylate buffer without KCl. ^1^H NMR spectra were then recorded at different temperatures between 278K and 303K in the presence of 100 mM KCl. (**C**) NMR titration with RHPS4. NMR measurements were conducted at 600 MHz and 293K in a buffer consisting of 20 mM cacodylate buffer, 100 mM KCl, and 10% D_2_O (NS 256). The imino proton region of the ^1^H NMR spectra of Pf_mtDNA_G4_1 was recorded with increasing concentrations of RHPS4 (12 mM stock solution in 62% DMSO). The RHPS4/DNA ratios are indicated on the left side of the spectra. (**D**) ITC-binding isotherm obtained at 20°C by titrating RHPS4 (200 mM concentration) to Pf_mtDNA_G4_1 (15 mM concentration). Both compounds were prepared in a buffer supplemented with 2% DMSO. Both the primary raw data (top) and the integrated heat data (bottom) are shown. (**E**) Thermal stabilization of the F-Pf_mtDNA-G4-1-T oligonucleotide measured by melting FRET assays. G4 ligands were added to the reaction at five molar equivalents (0.4 µM) with respect to F-Pf_mtDNA-G4-1-T (0.2 µM). The T1/2 value indicates the melting temperature of the oligonucleotide in different conditions.

In order to assess their folding *in vitro*, short DNA oligonucleotides were analyzed through biophysical methods. Eight sequences were first characterized using circular dichroism (CD). CD is a powerful and standard method for evaluating the folding properties of nucleic acids and characterizing G4 topologies (parallel-stranded and antiparallel-stranded G-tetrad cores) through distinct CD signatures. In the K^+^ conditions used, one of the eight sequences, Pf_mtDNA_G4_1, exhibited a CD profile with a positive band at ≈295 nm and a negative signal at ≈260 nm, a profile similar to those encountered for anti-parallel G4s (Fig. 4A). To further characterize these sequences, UV spectroscopy analyses and fluorescence-based assays were performed. Isothermal differential spectra (IDS) and thermal differential spectra (TDS) are two simple spectroscopic methods to demonstrate G4 formation based on the comparison of absorbance spectra of the folded and unfolded forms (48, 49). These methods involve comparing spectra recorded under permissive conditions (K^+^ for IDS, 25°C for TDS) for G4 formation and non-permissive conditions (no salt or Li^+^ for IDS, 95°C for TDS). Under these conditions, IDS and TDS showed no significant absorbance changes at 295 nm for seven sequences out of the eight considered. Again, Pf_mtDNA_G4_1 sequence was an exception, with TDS and IDS spectra reminiscent of G4-forming sequences, in agreement with its folding into a G4 *in vitro* (Fig. S3A and B). Finally, iso-FRET was used to demonstrate G4 formation. Iso-FRET is an isothermal fluorescence-based method which evaluates the ability of DNA oligonucleotides to bind a highly selective G4 ligand, PhenDC3, under competitive conditions with a well-characterized G4-forming sequence (50). In this assay, a reduction in the fluorescence signal resulting from the displacement of PhenDC3 from the reporter sequence indicates the propensity of the tested sequence to fold into a G4 structure *in vitro* and act as a competitor for PhenDC3. As shown in Fig. S3C, Pf_mtDNA_G4_1 induced a significant reduction in fluorescence emission as compared with other sequences tested, consistent with its propensity to adopt a G4 structure *in vitro*. Overall, four independent methods (CD, TDS, IDS, iso-FRET) demonstrated the ability of Pf_mtDNA_G4_1 to adopt a quadruplex fold.

We then considered nuclear magnetic resonance (NMR) to evaluate the capacity of the sequences to fold into G4 structures. NMR can unambiguously indicate the presence of such structures by following imino-protons that resonate at unique downfield frequencies (10–12 ppm) when involved in G-tetrads and consequently in Hoogsteen hydrogen bond networks, compared with imino protons involved in Watson-Crick base pairing that resonate between 12 and 14 ppm. NMR experiments were first conducted on synthetic oligonucleotides at 278K in a lithium cacodylate buffer with 150 mM KCl. Out of the eight considered sequences, only two (Pf_mtDNA_G4_1 and 3) gave reasonably clear and well resolved imino proton resonances in the 10–12 ppm range (Fig. S3D). The 2D NOESY spectra recorded for sequences 1, 3 and 7 revealed the presence of several NOEs between guanine imino protons, validating the presence of G4 structures. For three sequences (Pf_mtDNA_G4_4, 5, and 6), significantly broadened signals were observed, suggesting extensive exchange, which prevented a clear validation of unique G4 structures. Finally, the spectra recorded for sequences Pf_mtDNA_G4_2, 7, and 8 showed a reduced number of imino signals at 10–12 ppm compared with those within the range of 12–14 ppm, suggesting a poor propensity to form G4 structures in solution.

We focused on the Pf_mtDNA_G4_1 sequence for which all our biophysical data suggested G4 folding. At 278K, the 1D spectrum showed the presence of several peaks at 11–12.5 ppm, confirming the propensity of this sequence to form G4 structures in solution. Addition of a final K^+^ concentration of 100 mM was required to obtain characteristic guanine imino protons' chemical shifts involved in G-tetrad, which confirms the necessary presence of cations for G-rich oligonucleotides to fold into G4 structures (Fig. 4B). In the presence of 100 mM KCl, although the NMR spectrum did not show clear and sharp imino signals, the count of more than eight imino protons within that range suggests the formation of at least a two-layer G4 structure. Additional peaks were found between 8 and 10 ppm, likely corresponding to hydrogen-bonded guanine amino protons within the guanine quartets (Fig. 4B). These peaks broadened with increasing temperature and disappeared above 293K, while the G4 imino signals remained observable even at 303K, reflecting the relative stability of the structure.

To test if RHPS4 is able to interact with the Pf_mtDNA_G4_1 sequence, titration was performed at 293K by recording 1D NMR spectra upon addition of increasing concentrations of RHPS4. Effects were visible after addition of very low amounts of ligand (Fig. 4C). Notably, all proton signals significantly broadened upon the addition of RHPS4, and several shifts were observed, revealing that RHPS4 affects the G-quartets. Beyond a 1:1 ratio, no further chemical shift changes were observed, indicating that the complex had fully formed. By contrast, the addition of RHPS4 to the Pf_mtDNA_G4_2 oligonucleotide, which showed no evidence of G4 folding in CD experiments, did not induce any chemical shift changes (Fig. S3E).

We then applied isothermal titration calorimetry (ITC) to extract thermodynamic parameters associated with the association of RHPS4 to the Pf_mtDNA_G4_1 sequence. We found that the binding reaction was an exothermic process exclusively guided by a favorable enthalpy (∆H = −14.1 kcal/mol^−1^) (Fig. 4D). The stoichiometry close to one suggests that the RHPS4 ligand interacts with one quadruplex in a 1:1 complex, which is fully consistent with our NMR data. Fitting the ITC data to a one-site binding model provided an association constant of 0.56 ± 0.08 × 10^6^ M^−1^ at 20°C, which corresponds to a dissociation constant (Kd) of 1.8 ± 0.3 µM, i.e., in the low micromolar range.

To confirm the interaction of RHPS4 with the G4 structure formed by the Pf_mtDNA-G4-1 sequence and to evaluate its ability to stabilize this structure *in vitro*, FRET melting assays were performed (51). In this assay, the interaction of RHPS4 with the G4 structure is monitored through the increase in the half-melting temperature (T₁/₂) of the double-labeled G4-forming oligonucleotide F-Pf_mtDNA-G4-1-T (fluorescein (F) as the fluorescent donor and TAMRA (T) as the acceptor, attached respectively to the 5′ and 3′ ends for FRET analysis). Under 100 mM K^+^ conditions, the addition of five molar equivalents of RHPS4 relative to the oligonucleotide concentration led to a marked increase in the half-melting temperature (T₁/₂) by 4.5°C compared with ligand-free conditions, thereby demonstrating the capacity of this compound to interact with and stabilize the structure formed by the Pf_mtDNA-G4-1 sequence. The increase in T₁/₂ observed in the presence of RHPS4 was of a similar magnitude to that obtained with the highly selective G4 ligand 360A (51) (ΔT₁/₂ = 7.5°C relative to control conditions) (Fig. 4E), confirming that the observed shifts in melting temperature are consistent with the stabilization of a G4 structure formed by this oligonucleotide.

### *In vitro* RHPS4 pressure did not select for resistance

**Fig 5:**
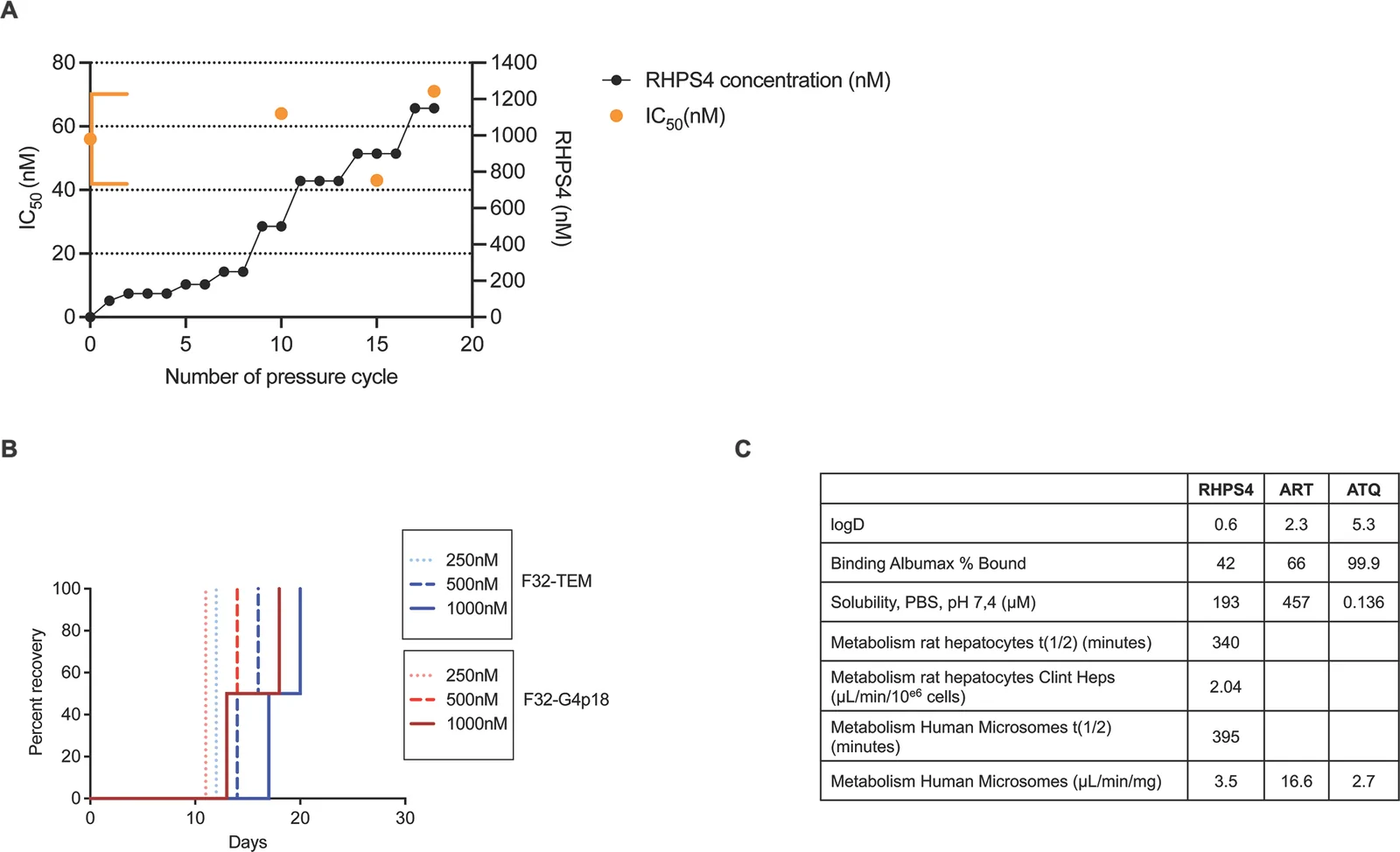
*In vitro* selection of RHPS4 pressure-adapted *Plasmodium falciparum* and pharmacological properties. (**A**) Evolution of RHPS4 concentration (dark line) over the course of 18-RHPS4 pressure cycles and IC_50_ values obtained for RHPS4 (orange) at cycle 0, 10, 15, and 18. (**B**) Kaplan-Meier analysis of *P. falciparum in vitro* recrudescence tests comparing the ability of the RHPS4-treated strain at cycle 18 (F32-G4p18, red color gradients) and the untreated control strain cultured in parallel (F32-TEM, blue color gradients) to proliferate after treatment with RHPS4 at 250, 500, and 1,000 nM. The Mantel-Cox test was used for statistical analysis of the recrudescence data performed in two independent experiments (**P < 0.001). (**C**) Pharmacological properties and metabolism data of RHPS4. Artemisinin (ART) and atovaquone (ATQ) were used as references; data from MMV.

One of the major challenges that new antiplasmodial drugs face upon implementation in the field is the appearance of drug-resistant *Plasmodium* parasites. To investigate the occurrence of such resistance for RHPS4, we submitted the F32-TEM parasite strain to *in vitro* discontinuous exposures of RHPS4 increasing concentrations from 90 nM up to 1.15 µM over a 17-month period. After 18 drug pressure cycles, no significant increase in the RHPS4 IC_50_ values was observed (Fig. 5A), with IC_50_ values between 64 nM and 71 nM. Moreover, there was no difference in recrudescence capacity between the selected parasite line obtained after the 18-drug pressures (F32-G4p18) and the untreated control tween strain cultured in parallel (F32-TEM), after 48 h of treatment with RHPS4 at concentrations of 250, 500, and 1,000 nM (corresponding to 3, 6, and 12 times the IC_50_; *P*-value = 0.08, 0.22, and 0.43 for 250 nM, 500 nM, and 1,000 nM, respectively; Fig. 5B). These results indicate that more than 1 year of RHPS4 pressure was not sufficient to select for resistance.

### Physico-chemical and pharmacological properties of RHPS4

Since RHPS4 activity, selectivity, and original mode of action make it a promising new antiplasmodial drug, we investigated some of its physico-chemical and metabolism properties. First, the value of LogD of 0.6 at pH 7.4 indicates that RHPS4 is more hydrophilic than ART (2.3) and ATQ (5.3) (52) (Fig. 5C). Then, with 42% binding to Albumax, RHPS4 displays lower interaction with human serum proteins, such as albumin, as compared with ART (66%) and ATQ (99.9%). Moreover, RHPS4 metabolization on rat hepatocytes and human microsomes is moderate and similar to ATQ, indicating good stability (Fig. 5C).

## DISCUSSION

We have demonstrated that RHPS4, a well-characterized G4 ligand, exhibits potent antiplasmodial activity (IC_50_ < 100 nM) against several strains of *Plasmodium falciparum*, including resistant laboratory strains and clinical isolates, with a high selectivity index (>100) relative to mammalian cells. These properties, combined with its favorable physicochemical and pharmacological profiles, position RHPS4 as a promising candidate for the development of novel antimalarial agents. A key advantage supporting its therapeutic potential is the absence of cross-resistance with front-line antimalarials such as chloroquine (CQ), atovaquone (ATQ), and artemisinin (ART). This suggests RHPS4 may be effective against multidrug-resistant malaria parasites, which are increasingly prevalent worldwide.

Stage-specific treatment assays revealed that RHPS4 exerts its antiparasitic effect primarily during the trophozoite stage (12–36 h post-invasion), a period marked by intense metabolic activity and active replication of both nuclear and mitochondrial DNA (53, 54). A similar stage-dependent profile has been described for ATQ, an established inhibitor of mitochondrial electron transport (46, 55). Pharmacodynamic analysis further showed that RHPS4 acts as a slow-acting compound, with a lag phase, parasite reduction ratio (PRR), and parasite clearance time (PCT) also comparable to those of ATQ. According to Sanz et al*.* (56), compounds with similar modes of action exert a similar killing profile. Therefore, we wondered if RHPS4 was also targeting the mitochondrion, as evidenced in mammalian cells at 1 µM (28). When used in *P. falciparum*, at fivefold higher concentration than the IC_50_ value (i.e., 450 nM, corresponding to the highest concentration tested to determine the moment of action in the parasite, Fig. 1E), we observed a pan-accumulation of RHPS4 in the whole parasite at the trophozoite stage, when RHPS4 is the most active. Interestingly, RHPS4 was not detectable at the ring stage and preferentially accumulated in the nuclei at the schizont stage, both stages when RHPS4 is the less active.

Molecular analyses revealed that RHPS4 significantly compromises mitochondrial DNA (mtDNA) integrity. Treatment with RHPS4 induced a 5- to 10-fold reduction in mtDNA abundance, compared with total DNA, across all *P. falciparum* strains tested, correlating with a marked decrease in transcript levels of mitochondrial-encoded genes. In contrast, treatment with ART or ATQ did not affect mtDNA abundance, indicating that RHPS4 likely acts through a novel mechanism involving the disruption of mitochondrial genome maintenance and transcription. Originally characterized as a telomerase inhibitor due to its ability to bind and stabilize human telomeric G4 structures, RHPS4 has been predominantly studied in the context of telomere dysfunction (34, 36, 37). However, work by Falabella and colleagues demonstrated a preferential activity of RHPS4 toward mitochondrial nucleic acids in mammalian cells. Compared with other G4 ligands with similar *in vitro* binding and stabilization capacities, RHPS4 exhibits mitochondrial localization in mammalian cells, likely due at least in part to its net positive charge, which may promote its accumulation within mitochondria via the organelle membrane potential (28).

Although our microscopy data did not reveal a specific accumulation of RHPS4 within parasite mitochondria, these results do not exclude the possibility that the compound penetrates this organelle. This hypothesis is supported by the observation that RHPS4 induces in *P. falciparum* a dramatic and selective reduction in both mitochondrial DNA (mtDNA) and mitochondrial RNA (mtRNA) (Fig. 3). Moreover, these effects occur predominantly during the trophozoite stage, when RHPS4 fluorescence is detected throughout the parasite. In contrast, no antiplasmodial activity is observed during the ring stage, when no RHPS4 fluorescence is detected, and only limited activity is observed at the schizont stage, where the fluorescence is largely restricted to the nuclear compartment (Fig. 2B; Fig. S1B). These observations are therefore consistent with mitochondrial access of RHPS4 during the trophozoite stage, despite the absence of a detectable mitochondrial-specific fluorescent signal.

It is worth noting that we did not observe any loss of apicoplast DNA (Fig. S2C), suggesting that the mitochondrial effects of RHPS4 do not result from a non-selective action on the parasites’ genetic material.

We then wondered if the observed loss of mtDNA could be linked to RHPS4’s interaction with mitochondrial G4s. Our bioinformatic and biophysical analyses identified at least one sequence within the *P. falciparum* mitochondrial genome capable of forming a two-tetrad G4 structure. Although such structures are inherently less stable than canonical three-tetrad G4s, our data indicate that it remains stable at 30°C, suggesting functional relevance under physiological conditions. Moreover, binding studies with RHPS4 and other G4 ligands support further stabilization of this structure, consistent with a potential role in mediating RHPS4’s mitochondrial effects. Although a direct interaction between RHPS4 and mitochondrial G4s cannot be formally (*in situ*) demonstrated, the effects of PhenDC3 and 360A (two of the most selective G4 ligands described to date) on mtDNA levels suggest a shared mechanism of action. It is worth noting that the effects of G4 ligands on *Plasmodium* mtDNA appear to depend on their overall net charge, as Pyridostatin and CX-5461, which lack a permanent net positive charge, show no significant effect on the parasite’s mtDNA. However, the marked differences in activity between RHPS4 and other positively charged G4 ligands suggest that additional factors contribute to their ability to disrupt the mitochondrial genome.

Altogether, these findings support a model in which RHPS4 binds G4 motifs within mtDNA in *P. falciparum*.

This mechanism distinguishes RHPS4 from existing antimalarial drugs, including the slow-acting ATQ compound, which targets the mitochondrial cytochrome bc1 complex without altering mtDNA abundance. The identification of Pf_mtDNA_G4_1 as a functional G4 motif provides, to our knowledge, the first direct evidence of a G-quadruplex structure in *P. falciparum* mitochondria. It suggests that mtDNA G4 structures could represent unexplored druggable targets for antimalarial strategies, particularly in the context of rising resistance to current treatments.

In conclusion, our study highlights RHPS4 as a promising antiplasmodial with a novel mechanism of action and showing no cross-resistance with other antimalarial compounds. Taken together, our data show that RHPS4 induces the death of the parasites through a process, not yet completely elucidated, that clearly involves the parasites’ mitochondria. Our results thus open new avenues for antimalarial drug discovery targeting mitochondrial nucleic acids and underscore the potential of G4 ligands in combating drug-resistant malaria.

## MATERIALS AND METHODS

### Drugs

Atovaquone (ATQ) and artemisinin (ART) were purchased from TCI Chemicals, chloroquine diphosphate (CQ) from Sigma Aldrich/Merck (Germany), and RHPS4 was purchased from MedChemExpress (MCE). Stock solutions were obtained by dissolving compounds in dimethyl sulfoxide (DMSO, Sigma-Aldrich) for ART, ATQ, and RHPS4, and in RPMI-1640 for CQ. The percentage of DMSO in all experiments is less than or equal to 0.5%.

### Human cell lines and culture conditions

All the cell lines used in this study were obtained from ATCC that certified their identity. DAPI staining analysis was used to confirm the absence of *Mycoplasma* contamination during the course of the study. All culture media were provided by Gibco and were supplemented with 10% fetal bovine serum (Eurobio), 100 U/mL penicillin (Gibco), and 100 µg/mL streptomycin (Gibco). HEK, HepG2, and BJ-hTert cells were grown in Dulbecco’s Modified Eagle Medium cells in a humidified atmosphere with 5% CO_2_ at 37°C.

### Parasite strains

F32-ART is a laboratory strain, obtained after artemisinin drug pressures and carrying the M476I mutation on the *pfk13* gene responsible for its ART resistance (3). F32-TEM is its twin ART-sensitive line from the strain F32-Tanzania (43). IPC8262 (*pfk13* C580Y, *pfcrt* M74I/N75E/K76T) is a field isolate from Cambodia resistant to both ART and CQ, provided by Dr. B. Witkowski of the Pasteur Institute in Cambodia. Q120 (*pfcytb* Y268N, *pfcrt* K76T) is a field isolate from Guyana resistant to both ATQ and CQ, provided by Dr. L. Musset from Pasteur Institute of Cayenne. The 3D7 lab strain is drug sensitive, Dd2 is CQ-resistant, and RF12 is both ART- and CQ-resistant (57).

### Parasite culture

Parasites were cultured according to Trager and Jensen (58) with slight modifications. All field isolates (IPC8262 and Q120) were cultured at 2% hematocrit in human red blood cells (EFS, French blood bank, France) in RPMI-1640 medium (with HEPES and L-glutamine, Dutscher, France) supplemented with 5% human serum (EFS, French blood bank, France), 0.55% Albumax II (Fisher Scientific, France), 0.4 mM hypoxanthine, 1 mM L-glutamine, and 11 µg/mL gentamicin, and parasites incubated with 5% O_2_, 5% CO_2_ and 90% N_2_ at 37°C. In parallel, the strains F32-TEM and F32-ART were cultivated at 2% hematocrit in human red blood cells in RPMI-1640 medium supplemented with 5% human serum and incubated with 5% CO_2_ at 37°C in humidified atmosphere.

### Chemosensitivity assay

The SYBR Green I assay was carried out to evaluate the *in vitro* antimalarial activity of the different compounds (59). D-sorbitol-synchronized ring-stage parasites were adjusted at 1% parasitemia before being exposed during 48 h at 37°C to different concentrations of the drugs in 96-well plates. Each concentration was tested in triplicate. Fluorescence was measured at 485-nm excitation/528-nm emission in the VICTOR Nivo plate reader (Perkin Elmer, USA). The IC_50_ values were then calculated using GraphPad Prism software (USA).

### Recrudescence assay

A recrudescence assay was performed on the strain sets: F32-ART vs F32-TEM, to compare the ability of each strain to survive and replicate after drug exposure (44). D-sorbitol-synchronized ring-stage parasites adjusted to 3% parasitemia and 2% hematocrit, were treated with the drug of interest in a 6-well plate. After 48 h of exposure, the drug was washed off with RPMI-1640, and the parasites were transferred to drug-free culture conditions at 10% human serum. Blood smears were made to follow recrudescence until the day that each parasite culture reached the 3% initial parasitemia, defined as the day of recrudescence. If no parasite recrudescence was observed after 30 days, the experiment was stopped, and data were censored. Data analysis was performed using Kaplan-Meier survival curves. Statistical significance was determined by a log-rank (Mantel-Cox) test using GraphPad Prism software (USA).

### Viability assay

Cells were seeded in 96-flat-well plates at 3,500 cells per well. Serial dilutions of various compounds were realized, allowing the same solvent concentration for each condition, and cells were treated 24 h after seeding. After 3 days, cells were fixed for 1 h at 4°C by addition of 10% trichloroacetic acid to a 3.33% final concentration, before being washed with tap water and dried overnight. Cells were stained by incubation for 30 min at room temperature in a 1% acetic acid solution containing 0.057% sulforhodamine B (SRB), then cells were washed with 1% acetic acid and dried overnight. Finally, 200 μL of a 10 mM Tris-base solution was added, plates were agitated for 1 h at room temperature, and SRB levels were measured by absorbance at 490 nm using μQuant microplate spectrophotometer (Bio-Tek Instruments). Percentages of cell viability are expressed after normalization relative to untreated cells.

The evaluation of RHPS4 cytotoxicity was also conducted, in three independent experiments, in Vero cells using the MTT [3-(4,5-dimethylthiazol-2-yl)-2,5-diphenyl tetrazolium bromide] method to quantify cell metabolic activity. Vero cells were seeded at 10,000^4^ cells per well, in 96-well plates for 24 h prior to being exposed for 48 h to the drug tested in duplicate from 5 nM to 50 μM. Subsequently, MTT was added to each well for 1 h, then DMSO was added in order that the formazan product was evaluated by determination of the absorbance at a wavelength of 540 nm. The CC_50_ (50% inhibitory concentration) was computed for each drug and cell line with the GraphPad Prism software using nonlinear regression to a four-parameter logistic curve.

### Stage-specific activity of RHPS4

To determine the time of action of RHPS4 during parasite erythrocytic development (60), the parasite cycle was subdivided into five distinct time windows: 0–12, 12–24, 24–36, 36–48, and 48–60 h to cover one and a quarter parasite cycle. The complete cycle condition (0–48 h) was performed as a control, and an extended condition (0–60 h) was added to assess the effect of the molecule on parasite reinvasion. Synchronized ring-stage F32-TEM parasites (0–6 h post-invasion, synchronized by Percoll/sorbitol treatment) adjusted to 0.5% parasitemia and 2% hematocrit were divided into 24-well plates. Wells were assigned to each time slot and treated with three concentrations of the test molecule corresponding to the value of IC_50_, 2× IC,_50_ and 5× IC_50_, while an untreated well served as a control. Each treatment lasted 12 h, and after washing to eliminate the molecule, the parasites were cultured in drug-free medium until the end of the last treatment. At the end of the 60 h, blood smears were prepared. In addition, SYBR Green labeling was performed for each condition, and the percentage inhibition was determined based on the amount of labeled DNA, which reflects parasite proliferation.

### *In vitro* parasite reduction ratio assay (PRR)

The aim of this test was to assess the parasite killing rate (PRR) in response to treatment. It was carried out in accordance with the method described by Sanz *et al.* (56) with slight modifications. For this test, highly synchronized ring-stage F32-TEM parasites (0–4 h post-invasion, synchronized by Percoll/sorbitol treatment) adjusted to 0.5% parasitemia and 2% hematocrit (corresponding to 1 × 10^6^ parasites/mL) were exposed in six-well plates to the compound to be tested at a concentration corresponding to 10 times its IC_50_. Every 24 h for up to 120 h, culture was washed with RPMI-1640, and an aliquot was transferred to a 96-well plate in which a serial dilution (1:3) was performed in uninfected RBC at 2% hematocrit. In the six-well plate, parasites were treated again with the compound to be tested and incubated until the next time point, 24 h later. The 96-well plates were maintained in culture for 3 weeks before SYBR Green labeling to determine the number of wells in which parasites proliferated. The number of parasites alive at each time point is determined using the following formula: X^n-1^; where X represents the serial dilution factor (here = 3), and n is the number of wells in which parasites grow. The log of (viable parasites + 1) is plotted *versus* time and used to determine the Log(PRR), which is the difference in the number of viable parasites at the end of the lag phase and 48 h later. The lag phase is defined as the time needed to reach the maximum speed of action. 99.9% parasite clearance time (PCT) is the time needed to decrease parasite number by three logs.

### Confocal microscopy of RHPS4-treated parasites

F32-TEM parasites were treated with RHPS4 using different conditions described below, as well as with Hoechst 33342 (Enzo) to stain DNA and 100 nM Mitotracker Deep Red FM (Invitrogen) to stain the mitochondrion, according to the manufacturer’s recommendations. For RHPS4, asynchronous or synchronous (0–3 h post-invasion) parasites were treated with either 90 nM for 45 h (*n* = 2, giving no fluorescence signal) or 450 nM for 30 min (*n* = 7), for 6 h (*n* = 1), or for 8 h (*n* = 1), giving the same fluorescence signal regardless of the duration of treatment. Live parasites were mounted between slide and coverslip in PBS containing 0.1% human serum, and imaged at oil immersion 60×, using a FV1000 confocal microscope (Olympus). Hoechst 33342 signal was measured using the 405-nm laser, RHPS4 using the 473-nm laser, and Mitotracker Deep Red FM using the 635-nm laser. Image acquisition was performed with Z-stacking to cover the entire infected red blood cell. Z-reconstruction was made with Fiji (ImageJ) using the Z-project MAX-intensity. Scale bar corresponds to 5 µm.

### *In vitro* potentiation test

The effect of ATQ on RHPS4 activity in F32-TEM parasites was determined by potentiation experiments, as described by Benoit-Vical *et al*. (61). D-sorbitol-synchronized ring-stage parasites adjusted to 1% parasitemia, and 2% hematocrit was exposed to different concentrations of the molecules alone (RHPS4 and ATQ) or to combinations of the two molecules in a 96-well plate. After 72-h incubation, cells were washed with PBS 1× three times and labeled with SYBR Green I 1× for 1 h. IC_50_ values for the single molecule and the combination are then calculated using GraphPad Prism software (USA). Isobolograms are constructed by plotting a pair of fractional IC_50_ (FIC) for each combination of RHPS4 and ATQ. Fractional IC_50_ for each molecule was calculated by dividing the IC_50_ value for the combination by the IC_50_ value for the molecule alone. The experiment was run three times with three technical replicates.

### Relative quantification of mitochondrial DNA (qPCR) and RNA (qRT-PCR)

Total genomic DNA was extracted directly from *P. falciparum*-infected RBC as described in the manufacturer’s user manual of the High Pure PCR Template Preparation Kit (Roche). To measure the relative abundance of mitochondrial DNA, we designed primers for mitochondrial sequences of interest (Table S1). Real-time PCR (qPCR) using SsoFast Eva Green Supermix (Bio-Rad), 10 ng/reaction DNA, and 300 nM primers in 10 μL of final reaction volume was performed in a thermal cycler (Bio-Rad) with the following parameters: 95°C for 2 min; 39 cycles of 95°C for 10 s, 60°C for 10 s, followed by a melting curve from 65 to 95°C. The relative abundance of mitochondrial DNA was calculated by the ΔΔCt method using the nuclear gene PF3D7_1371000, which encodes ribosomal RNA 18S, for normalization. To quantify the relative abundance of mRNA or rRNA by real-time quantitative polymerase chain reaction (qRT-PCR), total RNA was isolated from parasite pellets (after RBC lysis by saponin treatment) using the RNeasy Mini kit (Qiagen, Germantown, MD). Next, cDNA was synthesized using the iTaq Universal SYBR Green One-Step Kit (Bio-Rad) with specific primers (Table S1), 20 ng/reaction, and 300 nM primers in 10 μL of final reaction volume in a thermal cycler (Bio-Rad) with the following parameters: 50°C for 10 min, followed by 95°C for 1 min, and 39 cycles at 95°C for 10 s and 60°C for 30 s, followed by a melting curve from 65°C to 95°C. The relative abundance of RNA was calculated according to the ΔΔCt method using the nuclear gene PF3D7_1121300, which encodes for the protein tyrosine kinase-like TLK2, for normalization.

### Spectroscopy

For these studies, oligonucleotides were purchased from Sigma and used without further purification. Stock solutions were prepared at 100 μM strand concentration in pure water. All oligonucleotides were annealed in a lithium cacodylate buffer supplemented with 100 mM KCl, kept at 95°C for 5 min, and slowly cooled to room temperature before measurements. Circular dichroism (CD), differential absorbance spectra (IDS and TDS), and iso-FRET experiments were performed, as previously described (48–50). The experiments were performed at 25°C in a lithium cacodylate buffer in the presence of 100 mM potassium ions.

FRET-melting experiments were carried out in 96-well plates on a CFX96 real-time PCR equipment (Bio-Rad). After an initial stabilization at 20°C for 10 min, the temperature was increased by a 0.5°C step every minute until 99°C. The labeled oligonucleotide was dissolved in stock pH 7.2 buffer (10 mM lithium cacodylate, 90 mM lithium chloride) to 100 µM stock concentration and stored at −20°C. The melting experiments were performed at a final 0.2-µM strand concentration of oligonucleotide with 5 M equivalents of ligands relative to oligonucleotide concentration. The measurement buffer consisted of 10 mM lithium cacodylate pH 7.2 buffer, 100 mM potassium chloride. The F-Pf_mtDNA-G4-1-T (5′-FAM-**TGGATGGTGTTGGCTGGGC**-TAMRA-3′) was purchased from EUROGENTEC.

### NMR experiments

Purified oligonucleotides were purchased from Eurogentec. Unless specified, powders were resuspended into a 20 mM lithium cacodylate buffer with 100 mM KCl at pH 6.8. The oligonucleotides were heated to 95°C for 5 min and subsequently cooled down slowly to room temperature. For NMR titrations, RHPS4 powder was resuspended into deuterated DMSO to reach a final concentration of 12.5 mM in 62% DMSO. NMR experiments were recorded using Bruker 600 and 700 MHz spectrometers equipped with a cryoprobe.

### Isothermal titration calorimetry experiments

Isothermal titration calorimetry experiments (ITC) were performed at 10 and 20°C using a PEAT-ITC instrument (Malvern Panalytical). Both ligand and DNA solutions were diluted in a cacodylate buffer with 100 mM KCl and 2% DMSO to allow correct ligand solubility. Titration cell was filled with a solution of 20-30 μM oligonucleotide, while the syringe was loaded with a RHPS4 solution of 200–300 μM. Experiments consisted of a first injection followed by 18 injections of 4 s with a 180-s interval between the injections. Control dilution experiments were performed by injecting ligand solution into the buffer alone placed in the measurement cell. The ITC titrations were processed using the MicroCal Origin software.

### Pf-LDH test

*P. falciparum* 3D7 (from BEI resource) lactate dehydrogenase (Pf-LDH) growth inhibition assay was carried out as described in reference 62, with minor modifications in the culture state of inoculum (10%–15% parasitaemia with ≥80% rings). Data were normalized to percent growth inhibition with respect to positive (0.2% DMSO as 0% inhibition) and negative (mixture of 100 mM chloroquine and 100 mM atovaquone as 100% inhibition) controls.

### Study of metabolic stability in rat hepatocytes

A solution of the test compound in Krebs-Henseliet buffer solution (1 µM) was incubated in pooled rat hepatocytes (1 × 10^6^cells/mL) for 0, 15, 30, 45, 60, 75, and 90 min at 37°C (5% CO_2_, 95% relative humidity). The reaction was terminated with the addition of ice-cold acetonitrile containing system suitability standard at designated time points. The sample was centrifuged (4,200 rpm) for 20 min at 20°C, and the supernatant was half diluted in water and then analyzed by means of LC-MS/MS. % Parent compound remaining, half-life (T_1/2_) and clearance (CL_int,app_) were calculated using standard methodology. The experiment was carried out in duplicate. Diltiazem, 7-ethoxy coumarin, propranolol, and midazolam were used as reference standards.

### Metabolic stability study using human liver microsomes

A solution of the test compounds in phosphate buffer solution (1 µM) was incubated in pooled human liver microsomes (0.5 mg/mL) for 0, 5, 20, 30, 45, and 60 min at 37°C in the presence and absence of NADPH regeneration system (NRS). The reaction was terminated with the addition of ice-cold acetonitrile containing system suitability standard at designated time points. The sample was centrifuged (4,200 rpm) for 20 min at 20°C, and the supernatant was half diluted in water and then analyzed by means of LC-MS/MS.% Parent compound remaining, half-life (T_1/2_) and clearance (CL_int,app_) were calculated using standard methodology. The experiment was carried out in duplicate. Verapamil, diltiazem, phenacetin, and imipramine were used as reference standards.

### Selection of RHPS4-resistant strain *in vitro*

RHPS4 resistance was selected by sequential exposure of F32-Tanzania parasites to several cycles of drug pressure according to the method described by Witkowski et al., with slight modifications (43). This methodology and the *P. falciparum* parasite strain F32-Tanzania used were already successful for obtaining parasites resistant to artemisinin (4, 43). Briefly, D-sorbitol-synchronized ring-stage parasites adjusted to 3% parasitemia and 3% hematocrit (corresponding to 9 × 10^7^ parasites) were exposed to discontinuous and increasing concentrations of RHPS4 (from 90 nM to 1.15 µM). After each 48 h of incubation, the drug was washed off once with RPMI-1640, and the parasites were transferred to drug-free culture conditions at 10% human serum. Blood smears were made to follow recrudescence until the day that parasite culture reached its initial parasitemia, defined as the day of recrudescence. Parasite culture was then continued under normal conditions (5% human serum) until the next drug pressure cycle. This parasite culture under G4 drug pressure was called F32-G4. In parallel, a flask of parasites from F32-Tanzania, namely F32-TEM, was maintained in continuous culture under the same conditions but without any drug pressure as control. For selection experiments, parasites were grown with 5% O_2_, 5% CO_2_ and 90% N_2_ at 37°C.

### Statistical analyses

Unless otherwise specified, all results are based on at least three independent experiments. Statistical analyses were performed using GraphPad Prism software. In all figures, statistically significant differences between specified conditions are indicated by asterisks (*P < 0.05; **P < 0.01; ***P < 0.001; ****P < 0.0001). NS denotes a non-significant difference.

## SUPPLEMENTAL MATERIAL

Supplemental material aac.00144-26-s0001.pdf Fig. S1 to S3; Tables S1 and S2.

ASM does not own the copyrights to Supplemental Material that may be linked to, or accessed through, an article. The authors have granted ASM a non-exclusive, world-wide license to publish the Supplemental Material files. Please contact the corresponding author directly for reuse.

## References

[1] World Health Organization. 2025. World malaria report 2025. https://www.who.int/teams/global-malaria-programme/reports/world-malaria-report-2025.

[2] Daskum A, Chessed G, Qadeer MA, Mustapha T. 2021. Antimalarial chemotherapy, mechanisms of action and resistance to major antimalarial drugs in clinical use: a review. Microbes and Infectious Diseases 2:130–142. doi: 10.21608/mid.2020.43941.1064

[3] Ariey F, Witkowski B, Amaratunga C, Beghain J, Langlois A-C, Khim N, Kim S, Duru V, Bouchier C, Ma L, et al.. 2014. A molecular marker of artemisinin-resistant *Plasmodium falciparum* malaria. Nature 505:50–55. doi:10.1038/nature12876 PMC5007947 24352242

[4] Ward KE, Fidock DA, Bridgford JL. 2022. *Plasmodium falciparum* resistance to artemisinin-based combination therapies. Curr Opin Microbiol 69:102193. doi:10.1016/j.mib.2022.102193 PMC9847095 36007459

[5] Rosenthal PJ, Asua V, Conrad MD. 2024. Emergence, transmission dynamics and mechanisms of artemisinin partial resistance in malaria parasites in Africa. Nat Rev Microbiol 22:373–384. doi:10.1038/s41579-024-01008-2 38321292

[6] Siqueira-Neto JL, Wicht KJ, Chibale K, Burrows JN, Fidock DA, Winzeler EA. 2023. Antimalarial drug discovery: progress and approaches. Nat Rev Drug Discov 22:807–826. doi:10.1038/s41573-023-00772-9 PMC10543600 37652975

[7] Burge S, Parkinson GN, Hazel P, Todd AK, Neidle S. 2006. Quadruplex DNA: sequence, topology and structure. Nucleic Acids Res 34:5402–5415. doi:10.1093/nar/gkl655 PMC1636468 17012276

[8] Spiegel J, Adhikari S, Balasubramanian S. 2020. The structure and function of DNA G-quadruplexes. Trends Chem 2:123–136. doi:10.1016/j.trechm.2019.07.002 PMC7472594 32923997

[9] Yan MP, Wee CE, Yen KP, Stevens A, Wai LK. 2023. G-quadruplex ligands as therapeutic agents against cancer, neurological disorders and viral infections. Future Med Chem 15:1987–2009. doi:10.4155/fmc-2023-0202 37933551

[10] De Cian A, Grellier P, Mouray E, Depoix D, Bertrand H, Monchaud D, Teulade-Fichou M-P, Mergny J-L, Alberti P. 2008. *Plasmodium* telomeric sequences: structure, stability and quadruplex targeting by small compounds. Chembiochem 9:2730–2739. doi:10.1002/cbic.200800330 18924216

[11] Calvo EP, Wasserman M. 2016. G-Quadruplex ligands: potent inhibitors of telomerase activity and cell proliferation in *Plasmodium falciparum*. Mol Biochem Parasitol 207:33–38. doi:10.1016/j.molbiopara.2016.05.009 27217226

[12] Anas M, Sharma R, Dhamodharan V, Pradeepkumar PI, Manhas A, Srivastava K, Ahmed S, Kumar N. 2017. Investigating pharmacological targeting of G-quadruplexes in the human malaria parasite. Biochemistry 56:6691–6699. doi:10.1021/acs.biochem.7b00964 29182860

[13] Harris LM, Monsell KR, Noulin F, Famodimu MT, Smargiasso N, Damblon C, Horrocks P, Merrick CJ. 2018. G-quadruplex DNA motifs in the malaria parasite *Plasmodium falciparum* and their potential as novel antimalarial drug targets. Antimicrob Agents Chemother 62:e01828-17. doi:10.1128/AAC.01828-17 PMC5826154 29311059

[14] Gazanion E, Lacroix L, Alberti P, Gurung P, Wein S, Cheng M, Mergny J-L, Gomes AR, Lopez-Rubio J-J. 2020. Genome wide distribution of G-quadruplexes and their impact on gene expression in malaria parasites. PLoS Genet 16:e1008917. doi:10.1371/journal.pgen.1008917 PMC7365481 32628663

[15] Craven HM, Nettesheim G, Cicuta P, Blagborough AM, Merrick CJ. 2023. Effects of the G-quadruplex-binding drugs quarfloxin and CX-5461 on the malaria parasite *Plasmodium falciparum*. Int J Parasitol Drugs Drug Resist 23:106–119. doi:10.1016/j.ijpddr.2023.11.007 PMC10711401 38041930

[16] Murat P, Balasubramanian S. 2014. Existence and consequences of G-quadruplex structures in DNA. Current Opinion in Genetics & Development 25:22–29. doi:10.1016/j.gde.2013.10.012 24584093

[17] Varshney D, Spiegel J, Zyner K, Tannahill D, Balasubramanian S. 2020. The regulation and functions of DNA and RNA G-quadruplexes. Nat Rev Mol Cell Biol 21:459–474. doi:10.1038/s41580-020-0236-x PMC7115845 32313204

[18] Rodriguez R, Miller KM, Forment JV, Bradshaw CR, Nikan M, Britton S, Oelschlaegel T, Xhemalce B, Balasubramanian S, Jackson SP. 2012. Small-molecule-induced DNA damage identifies alternative DNA structures in human genes. Nat Chem Biol 8:301–310. doi:10.1038/nchembio.780 PMC3433707 22306580

[19] De Magis A, Manzo SG, Russo M, Marinello J, Morigi R, Sordet O, Capranico G. 2019. DNA damage and genome instability by G-quadruplex ligands are mediated by R loops in human cancer cells. Proc Natl Acad Sci USA 116:816–825. doi:10.1073/pnas.1810409116 PMC6338839 30591567

[20] Kruisselbrink E, Guryev V, Brouwer K, Pontier DB, Cuppen E, Tijsterman M. 2008. Mutagenic capacity of endogenous G4 DNA underlies genome instability in FANCJ-defective C. elegans. Curr Biol 18:900–905. doi:10.1016/j.cub.2008.05.013 18538569

[21] Pavlova AV, Kubareva EA, Monakhova MV, Zvereva MI, Dolinnaya NG. 2021. Impact of G-quadruplexes on the regulation of genome integrity, DNA damage and repair. Biomolecules 11:1284. doi:10.3390/biom11091284 PMC8472537 34572497

[22] van Wietmarschen N, Merzouk S, Halsema N, Spierings DCJ, Guryev V, Lansdorp PM. 2018. BLM helicase suppresses recombination at G-quadruplex motifs in transcribed genes. Nat Commun 9:271. doi:10.1038/s41467-017-02760-1 PMC5773480 29348659

[23] Wu Y, Shin-ya K, Brosh RMJ. 2008. FANCJ helicase defective in *Fanconia anemia* and breast cancer unwinds G-quadruplex DNA to defend genomic stability. Mol Cell Biol 28:4116–4128. doi:10.1128/MCB.02210-07 PMC2423121 18426915

[24] Duardo RC, Guerra F, Pepe S, Capranico G. 2023. Non-B DNA structures as a booster of genome instability. Biochimie 214:176–192. doi:10.1016/j.biochi.2023.07.002 37429410

[25] Dahal S, Siddiqua H, Katapadi VK, Iyer D, Raghavan SC. 2022. Characterization of G4 DNA formation in mitochondrial DNA and their potential role in mitochondrial genome instability. FEBS J 289:163–182. doi:10.1111/febs.16113 34228888

[26] Doimo M, Chaudhari N, Abrahamsson S, L’Hôte V, Nguyen TVH, Berner A, Ndi M, Abrahamsson A, Das RN, Aasumets K, Goffart S, Pohjoismäki JLO, López MD, Chorell E, Wanrooij S. 2023. Enhanced mitochondrial G-quadruplex formation impedes replication fork progression leading to mtDNA loss in human cells. Nucleic Acids Res 51:7392–7408. doi:10.1093/nar/gkad535 PMC10415151 37351621

[27] Huang W-C, Tseng T-Y, Chen Y-T, Chang C-C, Wang Z-F, Wang C-L, Hsu T-N, Li P-T, Chen C-T, Lin J-J, Lou P-J, Chang T-C. 2015. Direct evidence of mitochondrial G-quadruplex DNA by using fluorescent anti-cancer agents. Nucleic Acids Res 43:10102–10113. doi:10.1093/nar/gkv1061 PMC4666356 26487635

[28] Falabella M, Kolesar JE, Wallace C, de Jesus D, Sun L, Taguchi YV, Wang C, Wang T, Xiang IM, Alder JK, Maheshan R, Horne W, Turek-Herman J, Pagano PJ, St Croix CM, Sondheimer N, Yatsunyk LA, Johnson FB, Kaufman BA. 2019. G-quadruplex dynamics contribute to regulation of mitochondrial gene expression. Sci Rep 9:5605. doi:10.1038/s41598-019-41464-y PMC6447596 30944353

[29] Kuang K, Li C, Maksut F, Ghosh D, Vinck R, Wang M, Poupon J, Xiang R, Li W, Li F, Wang Z, Du J, Teulade-Fichou M-P, Gasser G, Bombard S, Jia T. 2024. A G-quadruplex-binding platinum complex induces cancer mitochondrial dysfunction through dual-targeting mitochondrial and nuclear G4 enriched genome. J Biomed Sci 31:50. doi:10.1186/s12929-024-01041-6 PMC11089687 38741159

[30] Gardner MJ, Hall N, Fung E, White O, Berriman M, Hyman RW, Carlton JM, Pain A, Nelson KE, Bowman S, et al.. 2002. Genome sequence of the human malaria parasite *Plasmodium falciparum*. Nature 419:498–511. doi:10.1038/nature01097 PMC3836256 12368864

[31] Bhartiya D, Chawla V, Ghosh S, Shankar R, Kumar N. 2016. Genome-wide regulatory dynamics of G-quadruplexes in human malaria parasite *Plasmodium falciparum*. Genomics 108:224–231. doi:10.1016/j.ygeno.2016.10.004 27789319

[32] Smargiasso N, Gabelica V, Damblon C, Rosu F, De Pauw E, Teulade-Fichou M-P, Rowe JA, Claessens A. 2009. Putative DNA G-quadruplex formation within the promoters of *Plasmodium falciparum* var genes. BMC Genomics 10:362. doi:10.1186/1471-2164-10-362 PMC2736202 19660104

[33] Stanton A, Harris LM, Graham G, Merrick CJ. 2016. Recombination events among virulence genes in malaria parasites are associated with G-quadruplex-forming DNA motifs. BMC Genomics 17:859. doi:10.1186/s12864-016-3183-3 PMC5093961 27809775

[34] Gowan SM, Heald R, Stevens MF, Kelland LR. 2001. Potent inhibition of telomerase by small-molecule pentacyclic acridines capable of interacting with G-quadruplexes. Mol Pharmacol 60:981–988. doi:10.1124/mol.60.5.981 11641426

[35] Leonetti C, Amodei S, D’Angelo C, Rizzo A, Benassi B, Antonelli A, Elli R, Stevens MFG, D’Incalci M, Zupi G, Biroccio A. 2004. Biological activity of the G-quadruplex ligand RHPS4 (3,11-difluoro-6,8,13-trimethyl-8H-quino[4,3,2-kl]acridinium methosulfate) is associated with telomere capping alteration. Mol Pharmacol 66:1138–1146. doi:10.1124/mol.104.001537 15304549

[36] Salvati E, Leonetti C, Rizzo A, Scarsella M, Mottolese M, Galati R, Sperduti I, Stevens MFG, D’Incalci M, Blasco M, Chiorino G, Bauwens S, Horard B, Gilson E, Stoppacciaro A, Zupi G, Biroccio A. 2007. Telomere damage induced by the G-quadruplex ligand RHPS4 has an antitumor effect. J Clin Invest 117:3236–3247. doi:10.1172/JCI32461 PMC2000812 17932567

[37] Rizzo A, Salvati E, Porru M, D’Angelo C, Stevens MF, D’Incalci M, Leonetti C, Gilson E, Zupi G, Biroccio A. 2009. Stabilization of quadruplex DNA perturbs telomere replication leading to the activation of an ATR-dependent ATM signaling pathway. Nucleic Acids Res 37:5353–5364. doi:10.1093/nar/gkp582 PMC2760797 19596811

[38] Granotier C, Pennarun G, Riou L, Hoffschir F, Gauthier LR, De Cian A, Gomez D, Mandine E, Riou J-F, Mergny J-L, Mailliet P, Dutrillaux B, Boussin FD. 2005. Preferential binding of a G-quadruplex ligand to human chromosome ends. Nucleic Acids Res 33:4182–4190. doi:10.1093/nar/gki722 PMC1181860 16052031

[39] De Cian A, Delemos E, Mergny J-L, Teulade-Fichou M-P, Monchaud D. 2007. Highly efficient G-quadruplex recognition by bisquinolinium compounds. J Am Chem Soc 129:1856–1857. doi:10.1021/ja067352b 17260991

[40] Rodriguez R, Müller S, Yeoman JA, Trentesaux C, Riou J-F, Balasubramanian S. 2008. A novel small molecule that alters shelterin integrity and triggers a DNA-damage response at telomeres. J Am Chem Soc 130:15758–15759. doi:10.1021/ja805615w PMC2746963 18975896

[41] Xu H, Di Antonio M, McKinney S, Mathew V, Ho B, O’Neil NJ, Santos ND, Silvester J, Wei V, Garcia J, et al.. 2017. CX-5461 is a DNA G-quadruplex stabilizer with selective lethality in BRCA1/2 deficient tumours. Nat Commun 8:14432. doi:10.1038/ncomms14432 PMC5321743 28211448

[42] Li Y, Nguyen M, Laurent M, Wein S, Paloque L, Kessavdjee-Djouma S, Witkowski B, Musset L, Augereau J-M, Cerdan R, Robert A, Liu Y, Meunier B, Benoit-Vical F. 2025. Emoquine-1: a hybrid molecule efficient against multidrug-resistant *Plasmodium* parasites, including the artemisinin-resistant quiescent stage, and also active *in vivo*. J Med Chem 68:3559–3571. doi:10.1021/acs.jmedchem.4c02702 39849946

[43] Witkowski B, Lelièvre J, López Barragán MJ, Laurent V, Su XZ, Berry A, Benoit-Vical F. 2010. Increased tolerance to artemisinin in Plasmodium falciparum is mediated by a quiescence mechanism. Antimicrob Agents Chemother 54:1872–1877. doi:10.1128/AAC.01636-09 PMC2863624 20160056

[44] Ménard S, Ben Haddou T, Ramadani AP, Ariey F, Iriart X, Beghain J, Bouchier C, Witkowski B, Berry A, Mercereau-Puijalon O, Benoit-Vical F. 2015. Induction of multidrug tolerance in *Plasmodium falciparum* by extended artemisinin pressure. Emerg Infect Dis 21:1733–1741. doi:10.3201/eid2110.150682 PMC4593447 26401601

[45] Gupta S, Thapar MM, Wernsdorfer WH, Björkman A. 2002. *In vitro* interactions of artemisinin with atovaquone, quinine, and mefloquine against *Plasmodium falciparum*. Antimicrob Agents Chemother 46:1510–1515. doi:10.1128/AAC.46.5.1510-1515.2002 PMC127123 11959589

[46] Nixon GL, Moss DM, Shone AE, Lalloo DG, Fisher N, O’Neill PM, Ward SA, Biagini GA. 2013. Antimalarial pharmacology and therapeutics of atovaquone. J Antimicrob Chemother 68:977–985. doi:10.1093/jac/dks504 PMC4344550 23292347

[47] Heald RA, Modi C, Cookson JC, Hutchinson I, Laughton CA, Gowan SM, Kelland LR, Stevens MFG, Acridines AP. 2002. Antitumor polycyclic acridines. 8.(1) synthesis and telomerase-inhibitory activity of methylated pentacyclic acridinium salts. J Med Chem 45:590–597. doi:10.1021/jm011015q 11806711

[48] Mergny J-L, Li J, Lacroix L, Amrane S, Chaires JB. 2005. Thermal difference spectra: a specific signature for nucleic acid structures. Nucleic Acids Res 33:e138. doi:10.1093/nar/gni134 PMC1201377 16157860

[49] Luo Y, Granzhan A, Marquevielle J, Cucchiarini A, Lacroix L, Amrane S, Verga D, Mergny J-L. 2023. Guidelines for G-quadruplexes: I. *in vitro* characterization. Biochimie 214:5–23. doi:10.1016/j.biochi.2022.12.019 36596406

[50] Luo Y, Verga D, Mergny J-L. 2022. Iso-FRET: an isothermal competition assay to analyze quadruplex formation *in vitro*. Nucleic Acids Res 50:e93–e93. doi:10.1093/nar/gkac465 PMC9458428 35670668

[51] De Cian A, Guittat L, Kaiser M, Saccà B, Amrane S, Bourdoncle A, Alberti P, Teulade-Fichou M-P, Lacroix L, Mergny J-L. 2007. Fluorescence-based melting assays for studying quadruplex ligands. Methods 42:183–195. doi:10.1016/j.ymeth.2006.10.004 17472900

[52] Freeman-Cook KD, Hoffman RL, Johnson TW. 2013. Lipophilic efficiency: the most important efficiency metric in medicinal chemistry. Future Med Chem 5:113–115. doi:10.4155/fmc.12.208 23360135

[53] Preiser PR, Wilson RJ, Moore PW, McCready S, Hajibagheri MA, Blight KJ, Strath M, Williamson DH. 1996. Recombination associated with replication of malarial mitochondrial DNA. EMBO J 15:684–693. PMC449987 8599952

[54] Inselburg J, Banyal HS. 1984. Synthesis of DNA during the asexual cycle of *Plasmodium falciparum* in culture. Mol Biochem Parasitol 10:79–87. doi:10.1016/0166-6851(84)90020-3 6420697

[55] Srivastava IK, Rottenberg H, Vaidya AB. 1997. Atovaquone, a broad spectrum antiparasitic drug, collapses mitochondrial membrane potential in a malarial parasite. J Biol Chem 272:3961–3966. doi:10.1074/jbc.272.7.3961 9020100

[56] Sanz LM, Crespo B, De-Cózar C, Ding XC, Llergo JL, Burrows JN, García-Bustos JF, Gamo F-J. 2012. *P. falciparum in vitro* killing rates allow to discriminate between different antimalarial mode-of-action. PLoS One 7:e30949. doi:10.1371/journal.pone.0030949 PMC3285618 22383983

[57] Okombo J, Mok S, Qahash T, Yeo T, Bath J, Orchard LM, Owens E, Koo I, Albert I, Llinás M, Fidock DA. 2022. Piperaquine-resistant PfCRT mutations differentially impact drug transport, hemoglobin catabolism and parasite physiology in *Plasmodium falciparum* asexual blood stages. PLOS Pathog 18:e1010926. doi:10.1371/journal.ppat.1010926 PMC9645663 36306287

[58] Trager W, Jensen JB. 1976. Human malaria parasites in continuous culture. Science 193:673–675. doi:10.1126/science.781840 781840

[59] Smilkstein M, Sriwilaijaroen N, Kelly JX, Wilairat P, Riscoe M. 2004. Simple and Inexpensive fluorescence-based technique for high-throughput antimalarial drug screening. Antimicrob Agents Chemother 48:1803–1806. doi:10.1128/AAC.48.5.1803-1806.2004 PMC400546 15105138

[60] Benoit-Vical F, Lelièvre J, Berry A, Deymier C, Dechy-Cabaret O, Cazelles J, Loup C, Robert A, Magnaval J-F, Meunier B. 2007. Trioxaquines are new antimalarial agents active on all erythrocytic forms, including gametocytes. Antimicrob Agents Chemother 51:1463–1472. doi:10.1128/AAC.00967-06 PMC1855510 17242150

[61] Benoit-Vical F, Robert A, Meunier B. 2000. *In vitro* and *in vivo* potentiation of artemisinin and synthetic endoperoxide antimalarial drugs by metalloporphyrins. Antimicrob Agents Chemother 44:2836–2841. doi:10.1128/AAC.44.10.2836-2841.2000 PMC90158 10991867

[62] Gamo F-J, Sanz LM, Vidal J, de Cozar C, Alvarez E, Lavandera J-L, Vanderwall DE, Green DVS, Kumar V, Hasan S, Brown JR, Peishoff CE, Cardon LR, Garcia-Bustos JF. 2010. Thousands of chemical starting points for antimalarial lead identification. Nature 465:305–310. doi:10.1038/nature09107 20485427

