# Supplementary material for: G-quadruplex-binding ligand RHPS4 reduces Plasmodium falciparum mitochondrial DNA level providing a novel antimalarial strategy
Source: Antimicrob Agents Chemother. 2026 Oct 1;70(11):e00144-26. doi: 10.1128/aac.00144-26 (PMC13731234; doi:10.1128/aac.00144-26)
Supplement: Supplementary file 1 [file aac.00144-26-s0001.pdf]

# Supplementary Figures and Tables

## G-Quadruplex-Binding Ligand RHPS4 reduces *Plasmodium falciparum* mitochondrial DNA level Providing a Novel Antimalarial Strategy

Mariam Salim[1,2], Lucie Paloque[1,2,$], Flore Nardella[1,2,$], Thibaud Reyser[1,2], Michel Nguyen[1,2], Jean-Michel Augereau[1,2], Yu Luo[3,4], Sébastien Britton[1], Jean-Louis Mergny[3], Virginie Gervais[5], Françoise Benoit-Vical[1,2,*], Dennis Gomez[1,*]

**The PDF file includes:**

Figures S1 to S3
Tables S1 to S2

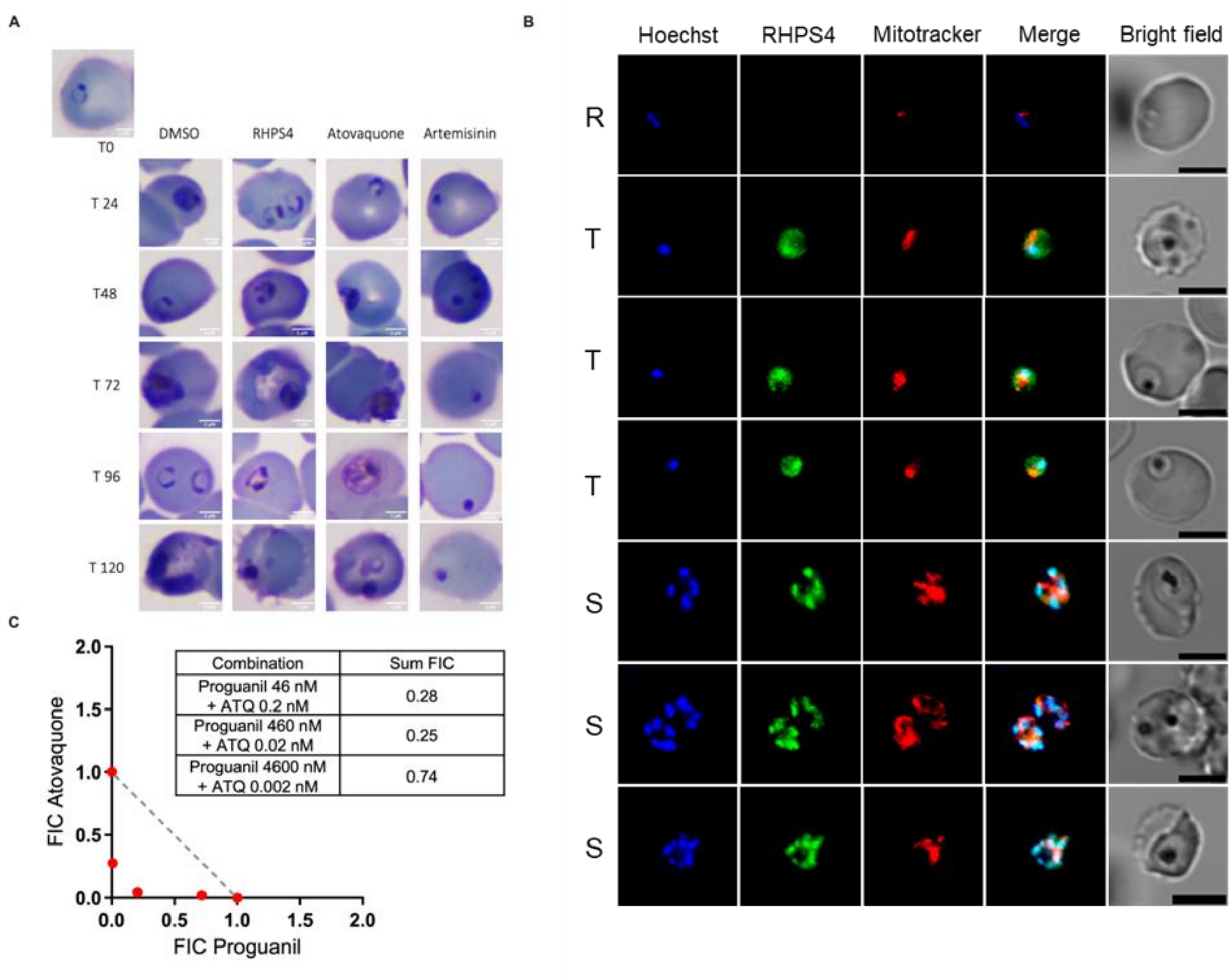


**Supplementary Figure S1: Effects of RHPS4 on *Plasmodium falciparum* morphology, RHPS4 cellular localization, and drug interaction compared with reference compounds.** **A** Giemsa smear showing the morphology over time of *P. falciparum* from a D-Sorbitol synchronized population after exposure to RHPS4 (900 nM), atovaquone (50 nM), and artemisinin (200 nM). The scale barin each picture corresponds to 2 µm. **B** Confocal microscopy images of parasites treated for 30 minutes with the nuclei acid strain Hoechst 33342 (blue), 450 nM RHPS4 (green) and 100 nM Mitotracker Deep Red FM (red) to label mitochondria at the stage of R = ring, T = trophozoite, S = schizont. Scale bar corresponds to 5 µm. **C** Isobologram and sum of fractional $IC_{50}$ (FIC) of interaction between atovaquone and proguanil (control combination) with *P. falciparum* F32-TEM strain.

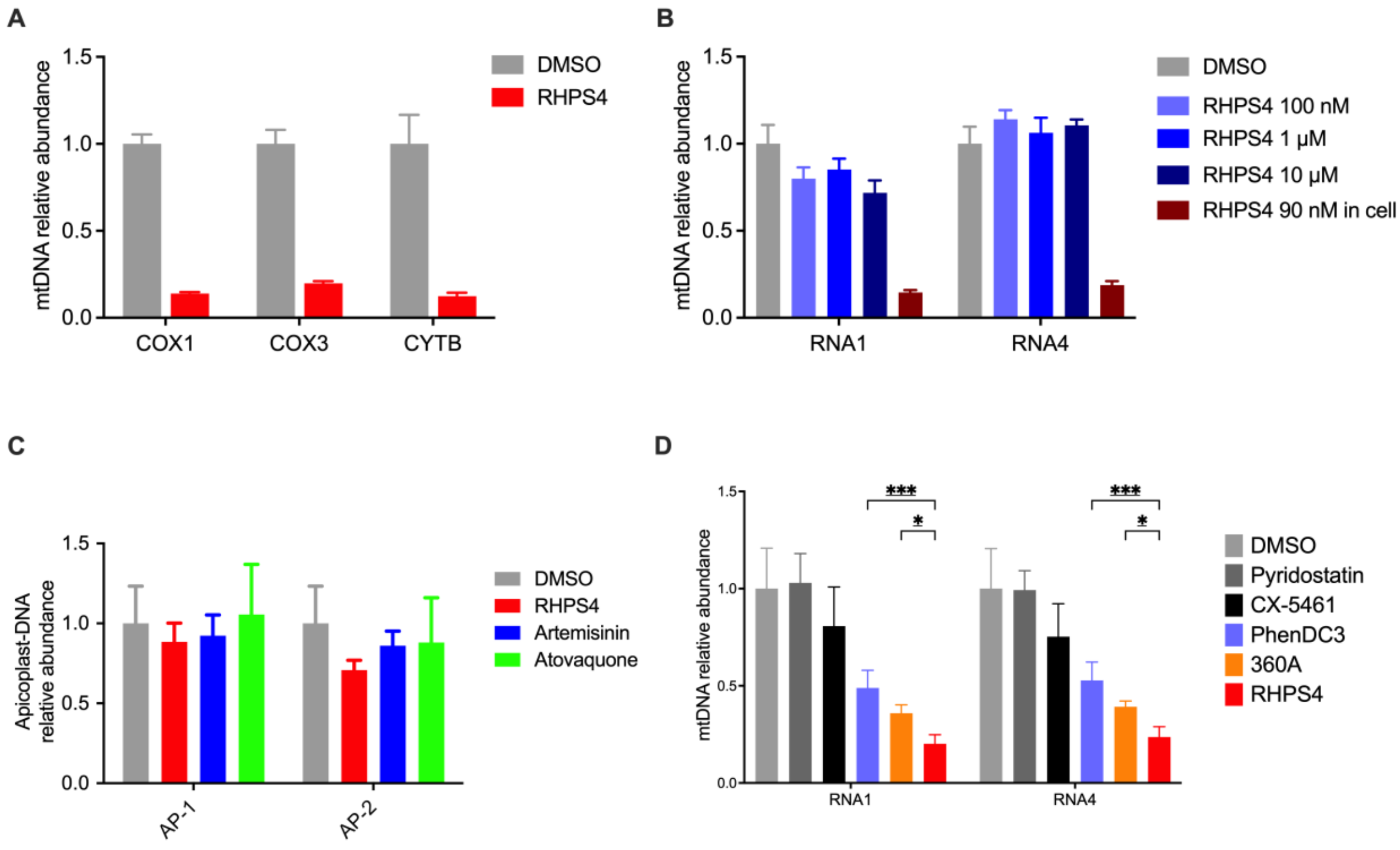


**Supplementary Figure S2: Effects of RHPS4 and G4 ligands on mitochondrial DNA abundance**. **A** Relative abundance of mtDNA sequences measured by qPCR after a 48-h exposure to 90 nM RHPS4 of trophozoite stage from F32-TEM (synchronized by sorbitol), n=1. The mtDNA abundance was normalized to the nuclear sequence PF3D7_1371000 coding for ribosomal RNA 18S. **B** Assessment of RHPS4 interference with the PCR reaction. The relative abundance of two mtDNA sequences extracted from untreated F32-TEM trophozoites were measured by qPCR in presence of different concentration of RHPS4. As a control, relative quantification of mtDNA sequences from RHPS4-treated parasites was performed in parallel, using the same PCR conditions. **C** Relative abundance of apicoplast DNA sequences measured by qPCR after a 48-h exposure to 90 nM RHPS4 (n=2). The apicoplast DNA abundance was assessed by qPCR assay targeting two regions of the apicoplast genome (sequences AP-1 and AP-2) and normalized to the nuclear sequence PF3D7_1371000 coding for ribosomal RNA 18S. **D** Differential effects of RHPS4 on mitochondrial DNA abundance compared to two other G4 ligands, 360A and PhenDC3**.** Relative abundance of mtDNA sequences measured by qPCR after 48-h of exposure to different G4 ligands at their respective $IC_{50}$ concentrations. mtDNA abundance was normalized to the nuclear gene PF3D7_1371000, which encodes 18S ribosomal RNA. Bars show mean ± SD from n = 3 independent experiments. *$p < 0.05$; ***$p < 0.001$.

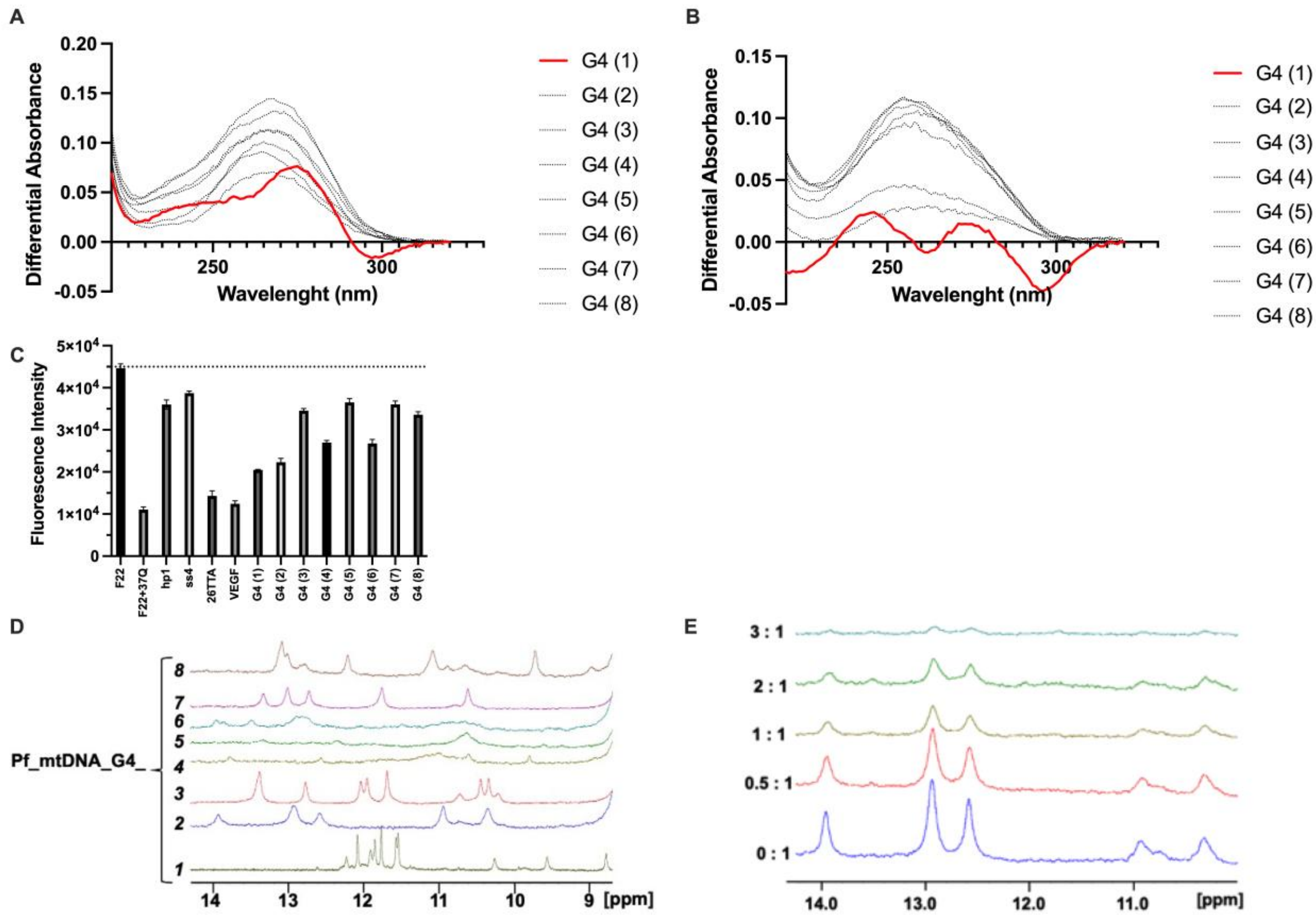


**Supplementary Figure S3: Biophysical characterization of G-quadruplex formation of mitochondrial sequences from *Plasmodium falciparum* A** TDS, **B** IDS and **C** iso-FRET results for the 8 sequences listed in Table 1 (in the same order). The only sequence for which CD/TDS/IDS results are clearly indicative of G4 formation is shown in red (Pf_mtDNA_G4_1). **D** Superimposition of the $^{1}$H NMR spectra recorded at 277 K for the eight considered sequences in a cacodylate buffer with 150 mM KCl at pH 6.5. **E** NMR Titration of Pf_mtDNA_G4_2 with RHPS4. NMR measurements were conducted at 600 MHz and 278 K in 20 mM cacodylate buffer, 100 mM KCl and 10 % $D_2O$ (256 scans). The imino proton regions of the $^{1}$H NMR spectra of Pf_mtDNA_G4_1 was recorded with increasing concentrations of RHPS4 (12 mM stock solution in 62 % DMSO). The RHPS4/DNA ratios are indicated on the left side of the spectra.

**Supplementary Table S1:** The gene name and ID and genome location, of the oligonucleotides used for qPCR, and RT-qPCR as indicated in the PlasmoDB database.

| Gene ID and name | Genome Location | Oligonucleotides |
|---|---|---|
| **PF3D7_MIT00900**<br>**Large subunit ribosomal RNA fragment G (LSUG)** | Pf3D7_MIT_v3:283..389(-) | 5′-GGGAACAAACTGCCTCAAGAC-3′<br>5′-GAGTGCGTAGCGAAGATTGC-3′ |
| **PF3D7_MIT01000**<br>**Small subunit ribosomal RNA fragment B (SSUB)** | Pf3D7_MIT_v3:390..505(-) | 5′-TGAAACAATGTGTTCCACCGC-3′<br>5′-ACGAAGATTGTAAGGTGAACGA-3′ |
| **PF3D7_MIT01100**<br>**Ribosomal RNA fragment 1 (RNA1)** | Pf3D7_MIT_v3:506..593(-) | 5′-GACCGGTCAAAACGGAATCA-3′<br>5′-AGAGCGGTAAACTATCGCCA-3′ |
| **PF3D7_MIT01400**<br>**Cytochrome c oxidase subunit 3 (COX3)** | Pf3D7_MIT_v3:734..1,483(-) | 5′-TGTAGAGATGCAAACATTCTCCT-3′<br>5′-TTTGTACGTCAGTATGTACTACC-3′ |
| **PF3D7_MIT02000**<br>**Ribosomal RNA fragment 3 (RNA3)** | Pf3D7_MIT_v3:1,830..1,910(+) | 5′-AGATCGCGTACTTTGGACCG-3′<br>5′-TATGTACGTGCGTTGTCCAC-3′ |
| **PF3D7_MIT02100**<br>**Cytochrome c oxidase subunit 1 (COX1)** | Pf3D7_MIT_v3:1,945..3,471(+) | 5′-ACCGGTTTTAACTGGAGGAGTA-3′<br>5′-GGGTTGTAAACGTCCTCTAGG-3′ |
| **PF3D7_MIT02300**<br>**Cytochrome b (CYTB)** | Pf3D7_MIT_v3:3,492..4,622(+) | 5′-AGCAAGTCGATATACACCAGATG-3′<br>5′-TATGTACGTGCGTTGTCCAC-3′ |
| **PF3D7_MIT02600**<br>**Ribosomal RNA fragment 4 (RNA4)** | Pf3D7_MIT_v3:4,624..4,698(+) | 5′-AGCGGAACAAATCAGACCGT -3′<br>5′-CTAAATGTACGAGTCGGCGG-3′ |
| **PF3D7_MIT03200**<br>**Large subunit ribosomal RNA fragment A (LSUA)** | Pf3D7_MIT_v3:5,026..5,201(-) | 5′-AGATTTCGCAGAAAACCGTCT-3′<br>5′-TTCTCAGGTTCCAAGCCAGA-3′ |
| **PF3D7_MIT04000**<br>**Large subunit ribosomal RNA fragment E (LSUE)** | Pf3D7_MIT_v3:5,577..5,771(-) | 5′-GTGCTCAGGGTCTTACCGTC-3′<br>5′-TTCTCAGGTTCCAAGCCAGA-3′ |
| **PF3D7_1121300**<br>**Tyrosine kinase-like protein (TKL2)** | Pf3D7_11_v3:801,809..808,198(-) | 5′-TCTTAAGCTCATGGAGGACAAA-3′<br>5′-CTTTACCACTCTTATACTTCCTTCC-3′ |
| **PF3D7_1371000**<br>**18S ribosomal RNA (18S)** | Pf3D7_13_v3:2,800,004..2,802,154(+) | 5′-TGCTTTTATTGCTTTTGAGAGGTTT-3′<br>5′-TGTCTGCCCATCAGTACTAACT-3′ |
| **PF3D7_API00600**<br>**tRNA Methionine** | Pf3D7_API_v3:1,034..1,120(+) | 5′-GGTCGAAAGCAATGGACTCA-3′<br>5′-ACGGACTCACCAATTTTCCTTAC-3′ |
| **PF3D7_API03600**<br>**Chaperone protein CIpM** | Pf3D7_API_v3:10,596..12,899(+) | 5′-TGGGCCTAGTGGTACTGGTAA-3<br>5′-AGTGGAGGACCAATACAACCA-3′ |

**Supplementary Table S2:** The gene name and ID, genome location of *P. falciparum* mitochondrial DNA sequences susceptible to G4 structuring as indicated in the PlasmoDB database.

| Sequence name | Gene ID and name | Genome Location |
|---|---|---|
| **Pf_mtDNA_G4_1** | **PF3D7_MIT00300 Ribosomal RNA fragment RNA9** | Pf3D7_MIT_v3:72..125(+) |
| **Pf_mtDNA_G4_2** | **PF3D7_MIT01500 Large subunit ribosomal RNA fragment F** | Pf3D7_MIT_v3:1,515..1,578(+) |
| **Pf_mtDNA_G4_3** | **PF3D7_MIT02100 Cytochrome c oxidase subunit 1** | Pf3D7_MIT_v3:1,945..3,471(+) |
| **Pf_mtDNA_G4_4** | **PF3D7_MIT02100 Cytochrome c oxidase subunit 1** | Pf3D7_MIT_v3:1,945..3,471(+) |
| **Pf_mtDNA_G4_5** | **PF3D7_MIT02100 Cytochrome c oxidase subunit 1** | Pf3D7_MIT_v3:1,945..3,471(+) |
| **Pf_mtDNA_G4_6** | **PF3D7_MIT04000 Large subunit ribosomal RNA fragment E** | Pf3D7_MIT_v3:5,577..5,771(-) |
| **Pf_mtDNA_G4_7** | **PF3D7_MIT04000 Large subunit ribosomal RNA fragment E** | Pf3D7_MIT_v3:5,577..5,771(-) |
| **Pf_mtDNA_G4_8** | **PF3D7_MIT04200 Ribosomal RNA fragment RNA8** | Pf3D7_MIT_v3:5,861..5,954(-) |
